# Plasma apolipoprotein E levels, isoform composition, and dimer profile in relation to plasma lipids in racially diverse patients with Alzheimer’s disease and mild cognitive impairment

**DOI:** 10.1186/s13195-023-01262-1

**Published:** 2023-07-03

**Authors:** Andreas Giannisis, Asma Al-Grety, Henrik Carlsson, Jennifer C. Howell, William T. Hu, Kim Kultima, Henrietta M. Nielsen

**Affiliations:** 1 Department of Biochemistry and Biophysics, Stockholm University, Svante Arrhenius Väg 16B, 114 18 Stockholm, Sweden; 2 Department of Medical Sciences, Clinical Chemistry, Uppsala University, Uppsala, Sweden; 3 Department of Neurology, Emory University, Atlanta, GA USA; 4 Department of Neurology, Rutgers-Robert Wood Johnson Medical School, and Institute for Health, Health Care Policy, and Aging Research, New Brunswick, NJ USA

**Keywords:** Black/African-Americans, Plasma, Apolipoprotein E, *APOEε4*, Alzheimer’s disease

## Abstract

**Background:**

The *APOE*ε4-promoted risk of Alzheimer’s disease (AD) is lower in Black/African-Americans (B/AAs), compared to non-Hispanic whites (NHWs). Previous studies reported lower plasma apolipoprotein E (apoE) levels in NHW *APOE*ε4-carriers compared to non-carriers, and low plasma apoE levels were directly associated with an increased risk of AD and all dementia. We further showed that *APOE*ε3/ε3 AD patients exhibited reduced plasma apoE dimers compared to corresponding control subjects. Whether plasma apoE levels and apoE dimer formation differ between races/ethnicities and therefore may help explain AD risk racial disparity remains to be elucidated.

**Methods:**

Using mass spectrometry, we determined total plasma apoE and apoE isoform levels in a cohort of B/AAs (*n* = 58) and NHWs (*n* = 67) including subjects with normal cognition (B/AA: *n* = 25, NHW: *n* = 28), mild cognitive impairment (MCI) (B/AA: *n* = 24, NHW: *n* = 24), or AD dementia (B/AA: *n* = 9, NHW: *n* = 15). Additionally, we used non-reducing western blot analysis to assess the distribution of plasma apoE into monomers/disulfide-linked dimers. Plasma total apoE, apoE isoform levels, and % apoE monomers/dimers were assessed for correlations with cognition, cerebrospinal fluid (CSF) AD biomarkers, sTREM2, neurofilament light protein (NfL), and plasma lipids.

**Results:**

Plasma apoE was predominantly monomeric in both racial groups and the monomer/dimer distribution was not affected by disease status, or correlated with CSF AD biomarkers, but associated with plasma lipids. Plasma total apoE levels were not related to disease status and only in the NHW subjects we observed lower plasma apoE levels in the *APOEε*4/ε4-carriers. Total plasma apoE levels were 2.6-fold higher in B/AA compared to NHW *APOE*ε4/ε4 subjects and associated with plasma high-density lipoprotein (HDL) in NHW subjects but with low-density lipoprotein levels (LDL) in the B/AA subjects. Higher plasma apoE4 levels, exclusively in *APOE*ε3/ε4 B/AA subjects, were linked to higher plasma total cholesterol and LDL levels. In the controls, NHWs and B/AAs exhibited opposite associations between plasma apoE and CSF t-tau.

**Conclusions:**

The previously reported lower *APOE*ε4-promoted risk of AD in B/AA subjects may be associated with differences in plasma apoE levels and lipoprotein association. Whether differences in plasma apoE levels between races/ethnicities result from altered *APOE*ε4 expression or turnover, needs further elucidation.

**Supplementary Information:**

The online version contains supplementary material available at 10.1186/s13195-023-01262-1.

## Background

According to the World Health Organization (WHO), Alzheimer’s disease (AD) is the most common form of neurodegenerative dementia affecting nearly 50 million people worldwide with higher incidence rates as age increases [1]. Among various described genetic risk factors [2], the ε4 allele of the *APOE* gene (*APOE* ε4) confers the strongest genetic risk to develop AD and dementia with Lewy bodies (DLB) [3]. Apolipoprotein E (apoE) is a 34-kDa glycoprotein, produced in the brain mainly by astrocytes [4] whereas in the periphery, more than 90% of the circulating apoE is derived from hepatocytes [5]. In humans, the *APOE* gene is polymorphic and exists in three common variants: ε2, ε3, and ε4 [6], where the ε3 allele is far more common than the ε2 and ε4 variants [7]. Harboring the ε4 allele is linked to a higher risk of AD and a younger age of disease onset [8]. Although the mechanisms that promote the increased risk of AD risk are not fully understood, various mechanisms suggesting loss of apoE neuroprotective function or a gain of neurotoxic function through amyloid-β (Αβ) dependent and/or independent cascades were suggested [9]. Despite the established connection between the ε4 variant and AD, cerebrospinal fluid (CSF) levels of total apoE and apoE isoforms appear not to be altered in AD patients versus controls nor differ between subjects with different *APOE* genotypes [10, 11]. Instead, low plasma apoE levels were shown to be directly associated with an increased risk of AD and all types of dementias, whereas higher levels appeared protective [12]. We and others have further documented that the presence of ε4 was associated with lower levels of total plasma apoE, which was mainly due to a specific reduction of the apoE4 isoform [10, 13, 14]. Interestingly, although plasma apoE is unable to cross the blood–brain barrier [5], we have also found that a higher ratio of plasma apoE4 to apoE3 levels was linked to negative brain imaging findings including gray matter atrophy and lower cerebral glucose metabolism [15], worse cognition, and more pathological CSF AD biomarker levels [16]. In a mouse model with humanized livers, we recently demonstrated that carrying ε4 specifically in the liver was associated with various pathological changes in the brain, and plasma apoE4 levels were significantly and negatively correlated with various synaptic marker levels in the hippocampus [17].

Despite the well-documented connection between *APOE* ε4 and neuropathological processes implicated in AD [18], it is important to highlight that also non-ε4 carriers develop AD which illustrates that other factors (i.e., environment, race/ethnicity, sex) may independently or synergistically promote or modify the risk of AD. There is a higher prevalence of the ε4 allele in Black/African American (B/AA) than non-Hispanic white (NHW) adults [19], which was previously discussed to partially explain the higher incidence of AD in older B/AA individuals [20–23]. However, subsequent studies found that B/AA ε4 carriers are at a relatively lower risk of developing AD than NHW ε4 carriers [24–26], possibly due to a protective variant (rs10423769) located on chromosome 19 [27]. More recently, it was proposed that ε4 ancestry (European compared to African local genetic ancestry) influenced *APOE* ε4 expression in the brain and that this expression difference may underlie previously documented variation in ε4-induced AD risk between populations of different races/ethnicities [28]. Even though the ε4-promoted risk of AD appears to be lower in B/AA adults, there is a higher general risk of AD and cognitive impairment in this population compared to NHW individuals [29–31].

Overall, since low plasma apoE levels were associated with a higher risk of AD [12] and specifically the *APOE* ε4 genotype appears to contribute to this higher risk by its association with lower plasma apoE levels [10, 32], we hypothesized that the difference in AD risk between populations might be attributed to corresponding differences in their plasma apoE levels, hence individuals with higher AD risk exhibiting lower plasma apoE levels and vice versa. In the current study, we aimed to assess the plasma apoE levels (total and isoform levels) in a cohort of older B/AA and NHW participants with normal cognition, mild cognitive impairment (MCI), or mild AD dementia with detailed neuropsychological, CSF, and magnetic resonance imaging (MRI) analysis [33]. Our main objective was to test whether, similar to NHW ε4 carriers, B/AA ε4-carriers also exhibit lower plasma apoE levels than non-carriers [10, 13, 14, 16]. Furthermore, we aimed to assess potential associations between plasma apoE levels, global cognition, AD CSF biomarkers (amyloid-β40 (Aβ_40_), amyloid-β42 (Aβ_42_), total tau (t-tau), and phosphorylated tau at threonine (Thr) 181 (p-tau181)) as well as CSF levels of neurofilament light (NfL) chain, soluble TREM2 (sTREM2), and plasma lipids. Last, we investigated whether the distribution of plasma apoE monomers and dimers in non-ε4-carriers differed between the studied diagnostic and ethnic groups.

## Methods

### Study participants

Plasma samples from a total of *n* = 125 individuals (*n* = 58 B/AA and *n* = 67 NHW older adults), part of a cross-sectional cohort enrolled at Emory University Atlanta GA (USA) during the period July 1, 2013, to June 30, 2015, were analyzed. Plasma was obtained after centrifugation of whole blood collected in tubes containing ethylenediaminetetraacetic acid (EDTA) as an anticoagulant, at 2000 × *g* and 4 °C for 10 min. Samples were aliquoted and stored at − 80 °C until analyzed. Of the B/AA participants, *n* = 25 were cognitively healthy, whereas the rest fulfilled the criteria for MCI (*n* = 24) or AD (*n* = 9). Of the ΝΗWs, *n* = 28 were deemed cognitively healthy, *n* = 24 were diagnosed with MCI, and *n* = 15 were AD patients. Race/ethnicity was ascertained through self-identification as previously described [34]. A diagnosis of MCI and AD dementia was made in accordance with the National Institute on Aging-Alzheimer’s Association guidelines [35]. Disease status, education, Mini-Mental State Examination (MMSE) scores, *APOE* genotype, and CSF levels of Αβ_42_, Αβ_40_, t-tau, p-tau, and NfL were previously described [33]. Levels of sTREM2 were recently reported in a study by Hu and colleagues [36]. A diagnosis of hyperlipidemia was present in *n* = 59 subjects (*n* = 28 B/AAs and *n* = 31 NHWs), and all were treated with statins. Levels of plasma triglycerides, total cholesterol, HDL, and LDL were assessed in routine by the Karolinska University Laboratory at the Karolinska University Hospital in Solna (Sweden).

### Sample preparation for LC–MS apoE analysis

Fifteen microliters of 100-fold diluted plasma samples in 50 mM ammonium bicarbonate were mixed 1:1 with 0.1% RapiGest SF Surfactant (Waters Corporation, Milford, MA, USA). Samples underwent reduction with 10 mM dithiothreitol (60 °C for 45 min, DTT, Sigma Aldrich, St. Louis, MO, USA), alkylation with iodoacetamide (in dark at room temperature for 40 min, IAA, Sigma Aldrich), and quenching with 20 mM DTT (at room temperature for 15 min). Quenched samples were then spiked with a mixture of heavy labeled peptides (SpikeTides TQL, JPT Peptide Technologies GmbH, Berlin, Germany, Additional file 1: Table S1) and digested with trypsin (0.1 mg/mL, Thermo Fisher Scientific, Waltham, MA, USA) followed by trypsin/lysine C (0.1 mg/mL, Thermo Fisher Scientific) each in a 1:20 ratio of enzyme to total protein. To increase the digestion efficiency, peptide digestion was performed in two consecutive steps. In the first step, the samples were treated with trypsin for 4 h, while in the second, the samples were treated with both trypsin and trypsin/lysine C for 18–19 h. In both steps, the samples were incubated at 37 °C. The next day, RapiGest SF surfactant was precipitated with 1% trifluoroacetic acid (TFA) and centrifuged at 17,000 × *g* for 15 min. The supernatant containing the digested peptides was collected, and the digested peptides were isolated by solid phase extraction using an Oasis hydrophilic-lipophilic balance (HLB) 96-well µElution Plate (2 mg Sorbent per well, 30 µm particle size, Waters) according to supplier’s guidelines. Modifications in the protocol were made in the washing step of the plate, in which MiliQ water was used, and in the elution step which was done with 25 µL of 100% methanol. Eluted peptides were dried under a vacuum and stored at − 80 °C until the day of analysis.

### Liquid chromatography–mass spectrometry apoE analysis

Liquid chromatography–mass spectrometry (LC–MS) analysis was performed essentially as previously described [15, 32, 37]. Dried digested apoE peptides were re-suspended in 30 µL of 0.1% formic acid in MiliQ water (Mobile phase A) and transferred in 0.3-mL polypropylene Snap Ring Micro-Vials (32 × 11.6 mm, Genetec, Montréal, Canada). Vials were sealed with polyethylene snap ring caps with a center hole (11 mm, Genetec) and processed for analysis. ApoE isoforms were separated using the LC Dionex UltiMateTM 3000 RSLC nano (Thermo Fisher Scientific). Five microliters of each sample were loaded on a reversed-phase C18 trap column (5 × 0.3 mm, 5 μm, Thermo Fisher Scientific) and further eluted from a PepMap C18 analytical column set at 40 °C (150 × 0.15 mm, 2 μm, Thermo Fisher Scientific) coupled to an Easy Spray source (Thermo Fisher Scientific) at 1 μL/min flow rate by increasing the concentration of mobile phase B (0.1% formic acid in acetonitrile) to a maximum of 40%, over an 11-min gradient. Detection and quantification of apoE isoforms were done using the mass spectrometer Q Exactive Orbitrap operated at single-stage full-scan (Thermo Fisher Scientific). The scan range was between 470 and 621 m/z, the automatic gain control target (AGC) was set at 1 × 10^6^, and the maximum injection time was 100 ms. Corresponding apoE peptide peaks were obtained at 7 × 10^4^ resolution.

### Total apoE and apoE isoform levels assessment

Levels of total apoE were quantified based on the amount of the common apoE peptide LGPLVEQGR and the sum of the peptides corresponding to apoE isoforms. For the quantification of the endogenous apoE peptides LGPLVEQGR, LAVYQAGAR, CLAVYQAGAR, LGADMEDVCGR, and LGADMEDVR, area response ratios between the endogenous digested apoE variant and the corresponding internal standard were extracted. Then, their ratio was used for the quantification of the concentration of the endogenous apoE peptides. The concentration of each peptide in fmoles/μL was quantified by multiplying the extracted area ratio with the spiked amount of the heavy variant (Additional file 1: Table S1) and then dividing it with the initial sample volume used for the study (15 μL). The obtained value was multiplied by 100 (initial plasma dilution) and finally reported as μg/mL using the apoE molecular weight (34 kDa) (Additional file 1: Fig. S1a).

Apolipoprotein E isoform levels were determined based on the linearity with the common apoE peptide. Thus, levels of apoE3 in *APOE* ε3/ε3, as well as apoE4 in *APOE* ε4/ε4 were determined based on the peptide LAVYQAGAR (Additional file 1: Fig. 1b). For *APOE* ε3/ε4 individuals, levels of apoE3 were determined by the use of the peptide LGADMEDVCGR. The apoE4 isoform levels in the same samples were calculated by subtracting the amount of the apoE3 isoform, as determined by the use of the peptide LGADMEDVCGR, from the amount of the peptide LAVYQAGAR which is common to both apoE3 and apoE4 isoforms and which showed excellent linearity with the common apoE peptide LGPLVEQGR (Additional file 1: Fig. S1b-c). The apoE4 peptide was used only for assessing the apoE4 phenotype and not for quantification due to high variability (Additional file 1: Fig. S1d), in line with previous studies [11]. Lastly, for heterozygous *APOE* ε2 carriers, the peptide CLAVYQAGAR was used to quantify the levels of the apoE2 isoform, while the peptide LAVYQAGAR was used for the determination of the apoE3 or apoE4 isoform concentrations. Levels of plasma apoE, as determined by the common apoE isoform or by summarizing the individual apoE isoforms, were plotted against each other in order to validate the quantification (Additional file 1: Fig. S1e). The peptides used for quantification were also used for assessing the linearity of the method by spiking increasing amounts (1–1539 fmoles) of the heavy labeled peptides in a plasma pool containing all the endogenous apoE peptides. Calibration curves, generated using the weighted sum of squares (1/*X*^2^), showed a coefficient of determination (R^2^) > 0.99 (Additional file 1: Fig. S2). The amounts of quantified plasma apoE ranged between 2.6 and 1384 fmoles. The method showed excellent reproducibility with intra- and inter-assay variations of < 5% and < 15%, respectively, for all the peptides. Chromatographic peaks corresponding to endogenous and labeled peptides were obtained using Xcalibur version 4.4 (Thermo Fisher Scientific) and quantified using Trace Finder 5.1 (Thermo Fisher Scientific). Generation of calibration curves was accomplished by using the GraphPad Prism, version 9 (GraphPad Inc., La Jolla, CA, USA).

### Assessment of plasma apoE monomer/dimer profiles

Non-reducing SDS-PAGE followed by western blot analysis was utilized for the detection and quantification of plasma apoE monomers, dimers, and heterodimers, as previously described [16]. Briefly, plasma samples were diluted 20-fold in phosphate-buffered saline (PBS) at pH 7.4 (VWR, Radnor, PA, USA) and mixed with non-reducing SDS-PAGE 4 × Laemmli Sample Buffer (Bio-Rad, Hercules, CA, USA). Samples in a scrambled order were added, and plasma proteins were separated using 4–15% pre-cast polyacrylamide gels (Tris-Glycise-TGX, Bio-Rad). After separation, the proteins were transferred to the Immobilon-P polyvinylidene difluoride (PVDF) membranes (Merck KGaA, Darmstadt, Germany) using the Bio-Rad Trans-blot semi-dry system. The membranes were blocked with 1% w/v non-fat dry milk powder (AppliChem GmbH, Darmstadt, Germany) in Tris-buffered saline (TBS, 20mM Tris base and 150mM NaCl) with 0.05% Tween® 20 (VWR) (TBS-T) and incubated overnight with the mouse anti-human apoE antibody WUE-4 (1 µg/ml, Novus Biologicals, Littleton, CO, USA). ApoE antibody reactivity was visualized by using donkey anti-mouse horse radish peroxidase-conjugated secondary antibody diluted in TBS-T to a final concentration of 0.2 µg/mL (Thermo Fisher Scientific) and enhanced chemiluminescence (ECL) solution (Advansta, Bering Drive, San Jose, CA, USA). The membranes were imaged using the Chemidoc XRS + imaging system (Bio-Rad), and optical densities were quantified using the ImageJ open-source software. The % distribution of plasma apoE into monomers and dimers was determined relative to the percentage of total plasma apoE reactivity.

### Data analysis

Statistical analyses of the MS and western blot-generated results were performed using the SPSS Statistics 28 (IBM Corp, Chicago, IL, USA) and the JMP Pro statistical software version 15.0.0 (SAS Institute, Cary, NC, USA). The distribution of the data was assessed by the use of the Kolmogorov–Smirnov test for normality. Non-normally distributed data were log-transformed, and the distribution was re-assessed. Comparisons between the two groups were performed using the Student’s *t*-test or Mann–Whitney *U*-test based on the distribution of the residuals in each group. Comparisons between ≥ 3 groups were performed either using analysis of variances (ANOVA) or the Kruskal–Wallis test followed by post hoc testing with the Tukey HSD or Dunn test. Group comparisons were also performed with *APOE* genotype as a covariate using a linear regression model, in which each *APOE* genotype was assigned two dummy variables (0 and 1). Model significance was assessed using the Wald chi-square test. Multiple comparisons (*n*) were accounted for by the use of Bonferroni correction. Potential associations between plasma apoE levels, the distribution of apoE in monomers and dimers (%), age, cognition, and CSF AD-relevant markers were assessed using either the Pearson’s (*r*) test for normally distributed data or the Spearman’s (*ρ*) test for non-normally distributed data. Correlations between plasma apoE levels with CSF AD biomarkers were assessed using partial correlations controlling for the *APOE* genotype (*r* (degrees of freedom)). The results are reported as average ± standard deviation or median (with range). A *p*-value of ≤ 0.05 was considered statistically significant.

## Results

### Study cohort demographics and clinical characteristics

Study subjects’ demographic and clinical characteristics, whereof some previously reported in detail [33, 36], are summarized in Table 1. Age, education, and levels of CSF sTREM2 were similar across the diagnostic groups. As expected, AD was associated with the lowest MMSE scores and the highest ratio of CSF t-tau/Aβ_42_ (Table 1). B/AAs and NHWs were of similar age, had similar years of education, and MMSE scores. NHW participants had higher levels of CSF sTREM2 than B/AA participants (*p* < 0.001, Mann–Whitney *U*-test), but sTREM2 levels did not differ between the diagnostic groups. The B/AA controls exhibited the lowest ε4 frequency (28%) compared to MCI (54%) and AD patients (89%) (chi-square, *p* = 0.006). Similarly, although not reaching statistical significance (chi-square, *p* = 0.101), the ε4 frequency in NHW controls was 39% versus 54% in MCI patients and 73% in AD patients. Detailed group comparisons of the CSF Αβ_40_, Αβ_42_, t-tau, and p-tau, and NfL levels as well as the t-tau/Αβ_42_ ratio were previously reported [33], and the data here were used only for the assessment of potential associations with levels of plasma apoE.

**Table 1:** Cohort demographics and clinical characteristics

Race/ethnicity	Diagnosis	*N* (females/males)	*APOE* ε4 (− / − , + / − , + / +)	Age (years)	Education (years)	MMSE score	CSF t-tau/Aβ_42_	CSF sTREM2 (pg/mL)
Whole cohort (*n* = 125)	Controls	53 (31/22)	35, 17, 1	69.2 ± 7.2	16.0 (10.0–22.0)	29.0 (24–30)	0.15 (0.06–3.09)	340.0 ± 123.7
	MCI	48 (25/23)	22, 21, 5	70.1 ± 6.7	16.0 (12.0–22.0)	27.0^*^ (22.0–30.0)	0.27 (0.06–3.4)	325.2 ± 104.6
	AD	24 (13/11)	5, 13, 6	68.6 ± 8.9	16.0 (12.0–22.0)	22.0^***,f^ (10.0–28.0)	0.86^***,f^ (0.17–3.84)	352.2 ± 126.8
	*p*-value	–^a^	0.001^a^	–^b^	–^c^	< 0.001^c^	< 0.001^c^	–^b^
B/AAs (*n* = 58)	Controls	25 (15/10)	18, 7, 0	67.4 ± 6.3	16.0 (10.0–22.0)	29.0 (24.0–30.0)	0.12 (0.07–0.51)	254.3 (109.7–446.7)
	MCI	24 (11/13)	11, 11, 2	68.9 ± 7.5	16.0 (12.0–22.0)	26.0 (22.0–30.0)	0.22 (0.06–3.40)	288.7 (101.2–449.3)
	AD	9 (5/4)	1, 6, 2	71.1 ± 10.2	18.0 (14.0–22.0)	21.0^***,f^ (10.0–25.0)	0.62^***^ (0.17–1.35)	249.3 (143.0–482.9)
	*p*-value	–^a^	0.014^a^	–^b^	–^c^	< 0.001^c^	< 0.001^c^	- ^b^
NHWs (*n* = 67)	Controls	28 (16/12)	17, 10, 1	70.9 ± 7.7	17.0 (12.0–22.0)	29 (27.0–30.0)	0.19 (0.06–3.09)	381.0 (256.9–658.8)
	MCI	24 (14/10)	11, 10, 3	71.3 ± 5.9	17.0^e^ (12.0–22.0)	28 (25.0–30.0)	0.41 (0.10–2.29)	356.3 (213.5–614.3)
	AD	15 (8/7)	4, 7, 4	67.1 ± 7.9	14.0^**^ (12.0–18.0)	22.0^***,f^ (15.0–28.0)	0.99^***,d^ (0.19–3.84)	366.9 (241.8–695.5)
	*p*-value	–^a^	–^a^	–^b^	0.002^c^	< 0.001^c^	< 0.001^c^	–^b^

Values are represented as average ± standard deviation or as median (minimum–maximum). *p*-values were obtained after comparisons between the diagnostic groups using ^a^chi-square, ^b^ANOVA, or ^c^Kruskal-Wallis. “–”: non-significant. *p ≤ 0.05, **p ≤ 0.01, and ***p ≤ 0.001 indicate comparison between MCI or AD patients and controls. ^d^*p* ≤ 0.05, ^e^*p* ≤ 0.01, ^f^*p* ≤ 0.001 indicate comparison between AD and MCI patients. *C*omparison between the diagnostic groups was performed by utilizing Dunn’s post hoc test followed by Bonferroni correction for multiple comparisons (*n* = 3)

*B/AAs* Black/African-Americans, *NHWs* Non-Hispanic whites, *MCI* Patients with mild cognitive impairment, *AD* Patients with Alzheimer’s disease, *APOE* Apolipoprotein E gene; − / − , + / − , + / + , *APOE* ε4 non-carriers, heterozygous, and homozygous, respectively, *MMSE* Mini-mental state examination score, *Aβ*_*42*_, amyloid-β42 peptide; *t-tau*, total tau, *sTREM2* soluble triggering receptor expressed on myeloid cells 2

### Plasma lipid levels

Plasma lipids levels in each diagnostic group are presented in Table 2. Classification of the study subjects based on their disease status, sex, race/ethnicity, and *APOE* ε4 genotype was used to assess whether any of these variables had any influence on the levels of plasma lipids. Where applicable, *APOE* genotype, sex, race/ethnicity, or education was used as co-factors in the analyses.

**Table 2:** Plasma lipid levels

Race/ethnicity	Diagnosis	TGs (mmol/L)	t-Ch (mmol/L)	LDL (mmol/L)	HDL (mmol/L)	LDL/HDL
Whole cohort (*n* = 125)	Controls (*n* = 53)	1.17 ± 0.61	4.71 ± 0.85	2.70 ± 0.78	1.49 ± 0.45	1.97 ± 0.77
	MCI (*n* = 48)	1.12 ± 0.40	4.60 ± 0.87	2.66 ± 0.80	1.46 ± 0.41	1.96 ± 0.83
	AD (*n* = 24)	1.22 ± 0.53	5.36 ± 1.16^*,**^	3.09 ± 0.81	1.73 ± 0.66	1.96 ± 0.79
	*p*-value	–^a^	0.004^a^	–^a^	–^a^	–^a^
B/AAs (*n* = 58)	Controls (*n* = 25)	1.24 ± 0.57	4.79 ± 0.91	2.81 ± 0.81	1.41 ± 0.38	2.12 ± 0.83
	MCI (*n* = 24)	1.00 ± 0.27	4.52 ± 0.90	2.66 ± 0.92	1.42 ± 0.42	2.07 ± 1.03
	AD (*n* = 9)	1.13 ± 0.44	5.43 ± 1.36	3.19 ± 0.68	1.74 ± 0.79	1.98 ± 0.58
	*p*-value	–^a^	–^a^	–^a^	–^a^	–^a^
NHWs (*n* = 67)	Controls (*n* = 28)	1.11 ± 0.66	4.64 ± 0.80	2.60 ± 0.74	1.55 ± 0.50	1.83 ± 0.70
	MCI (*n* = 24)	1.23 ± 0.48	4.69 ± 0.86	2.65 ± 0.68	1.50 ± 0.41	1.85 ± 0.58
	AD (*n* = 15)	1.27 ± 0.59	5.31 ± 1.07	3.03 ± 0.90	1.71 ± 0.61	1.95 ± 0.91
	*p*-value	–^a^	0.049^a^	–^a^	–^a^	–^b^

Values are represented as average ± standard deviation. *p*-values were obtained after comparisons between the diagnostic groups using ^a^ANOVA or ^b^Kruskal-Wallis. “–”: non-significant. *p ≤ 0.05 correspond to the comparison between AD patients and controls (95% CI: 0.11, 1.19), whereas **p ≤ 0.01 refers to the comparison between AD and MCI patients (95% CI: 0.21, 1.30) and acquired using the Tukey HSD test

*B/AAs* Black/African-Americans, *NHWs* Non-Hispanic whites, *MCI* Patients with mild cognitive impairment, *AD* Patients with Alzheimer’s disease, *TGs* Triglycerides, *t-Ch.* Total cholesterol, *LDL* Low-density lipoprotein, *HDL* High-density lipoprotein

In the whole cohort, patients with AD exhibited a 1.1-fold increase in plasma total cholesterol compared to controls (*p* = 0.016) and MCI patients (*p* = 0.004), with the latter significance remaining when controlling for *APOE* genotype (*p* = 0.013). Controlling for sex or education did not eliminate the significant difference in the plasma cholesterol levels between AD patients and MCI patients (*p*_education adjusted_ = 0.005, *p*_sex adjusted_ = 0.002), and controls (*p*_education adjusted_ = 0.017, *p*_sex adjusted_ = 0.006). Plasma triglycerides, LDL, HDL, and the LDL/HDL ratio did not differ between the diagnostic groups although B/AA MCI patients (*n* = 24) exhibited 23% lower plasma triglycerides compared to MCI NHW (Student’s *t*-test,* p* = 0.047, *n* = 24) (Table 2). After controlling for *APOE* genotype and education, the difference in the levels of plasma triglycerides between NHW and B/AA MCI patients remained (*p* = 0.039 and *p* = 0.031, respectively), however not when accounting for sex (*p* = 0.061).

Female subjects (*n* = 69), irrespective of diagnosis, had approximately 1.1, 1.6, and 1.2 times higher levels of plasma total cholesterol, HDL, and LDL compared to males (*n* = 56), respectively, with only the difference in total cholesterol remaining when stratifying the subjects based on race/ethnicity (Additional file 1: Fig. S4a-c). We also documented that B/AA females (*n* = 31) exhibited 28% lower levels of triglycerides compared to male B/AAs (*n* = 27) (Additional file 1: Fig. S4d). The observed differences in the levels of plasma lipids remained after accounting for the *APOE* genotype (Additional file 1: Fig. S4a-d). After controlling for years of education, the differences in plasma total cholesterol, LDL, and HDL remained (whole cohort: *p*_total cholesterol_ = 0.002, *p*_HDL_ = 0.003, *p*_LDL_ = 0.013; B/AAs: *p*_total cholesterol_ = 0.033, NHWs: *p*_total cholesterol_ = 0.028), whereas the difference in triglyceride levels (Additional file 1: Fig. S4d) was eliminated (*p* = 0.092).

Plasma total cholesterol and LDL levels were 1.1-fold higher in ε4 heterozygous (*n* = 51) individuals compared to non-carriers (*n* = 62, cholesterol: ANOVA, *p* = 0.019, Tukey HSD post hoc, *p* = 0.049; LDL: ANOVA, *p* = 0.018, Tukey HSD post hoc, *p* = 0.016). Despite the small sample size, plasma HDL levels were significantly influenced by *APOE* ε4 status (ANOVA, *p* = 0.040) with ε4 homozygous B/AA (*n* = 4) exhibiting 1.4-fold higher HDL compared to heterozygous ε4-carriers (*n* = 24, Tukey HSD post hoc, *p* = 0.034) and non-carriers (*n* = 30, Tukey HSD post hoc, *p* = 0.044). There was no race/ethnicity-based difference in plasma lipid levels between subjects of different *APOE* genotypes. Overall, the levels of total cholesterol and triglycerides appeared to differ between diagnostic and racial/ethnic groups, whereas sex and *APOE* ε4 status appeared to influence the levels of total cholesterol, HDL, and LDL.

### Plasma apoE phenotyping

Plasma apoE phenotyping was performed utilizing the combination of apoE endogenous peptides _104_LGADMEDVCGR_114_ (apoE2 and apoE3), _104_LGADMEDVR_112_ (apoE4), _159_LAVYQAGAR_167_ (apoE3 and apoE4), and _158_CLAVYQAGAR_167_ (apoE2) (Additional file 1: Table S2), based on the presence or absence of endogenous variants in the sample, as previously described [37]. The peptide _181_LGPLVEQGR_189_ which is common for all apoE isoforms was used only for quantification and not phenotyping. All the samples were run in a blinded manner with *APOE* genotypes unknown to the person performing the experiment. All assigned phenotypes were in 100% agreement with their previously determined *APOE* genotypes (Additional file 1: Table S3).

### Plasma apoE levels in relation to diagnosis, gender, *APOE* genotype, and race/ethnicity

Having quantified plasma apoE levels in all subjects, we further assessed whether plasma apoE levels varied due to disease status, comorbidities, sex, race/ethnicity, age, and *APOE* genotype. Where applicable, *APOE* genotype, sex, race/ethnicity, or education was used as co-factors in the analyses.

**Fig. 1:**
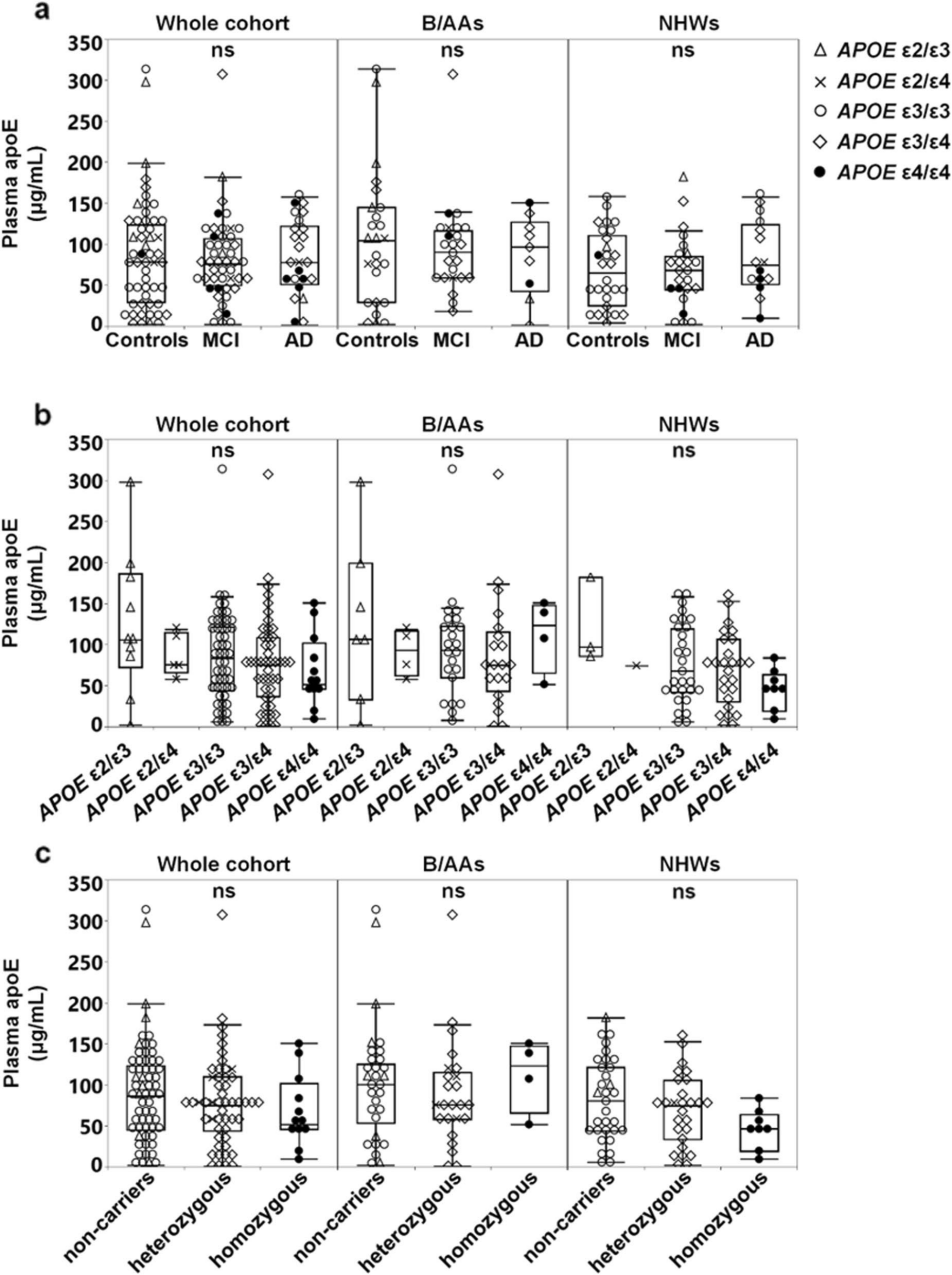
Plasma apoE levels in the different diagnostic groups and in study subjects with different *APOE* genotypes and race/ethnicities. **a** Plasma apoE levels between controls, as well as MCI and AD patients that were either Black/African-Americans (B/AAs) or non-Hispanic whites (NHWs). **b** Plasma apoE levels between subjects with *APOE* ε2/ε3, *APOE* ε2/ε4, *APOE* ε3/ε3, *APOE* ε3/ε4, and *APOE* ε4/ε4 genotypes. **c** Plasma apoE levels between subjects grouped based on the number of ε4 alleles. Data is presented in boxplots indicating the median, lower and upper quartile, and whiskers indicating the data range.

Plasma apoE levels did not differ across the diagnostic groups (*n* = 125, *p* = 0.892, Kruskal–Wallis, Fig. 1a), and this outcome did not change when accounting for race/ethnicity, *APOE* genotype, or their interaction (*APOE* genotype × race/ethnicity, *p* = 0.166). Regardless of diagnosis and race/ethnicity (*n* = 125), women had higher plasma total apoE levels than men (85.6 (0.60–313.7) μg/mL vs. 73.1 (2.11–173.2) μg/mL, Student’s *t*-test, *p* = 0.020) also when accounting for *APOE* genotype (Wald chi-square, *p* = 0.028). We found 40% higher plasma apoE levels in male B/AA (*n* = 27) compared to male NHW (*n* = 29) (Student’s *t*-test, *p* = 0.040); however, this difference was eliminated when taking *APOE* genotype into account (Wald chi-square, *p* = 0.069). On the contrary, plasma apoE levels did not differ between males and females with the same ethnic background (B/AA: Mann–Whitney *U*-test* p* = 0.198, NHW: Student’s *t*-test, *p* = 0.062) irrespective of *APOE* genotype.

Plasma apoE levels were not significantly associated with age (Pearson’s (*r*) =  − 0.094, *p* = 0.295) or education (Spearman’s (*ρ*) =  − 0.032, *p* = 0.726) and did not vary based on *APOE* genotype in the whole study cohort (*n* = 125), or across races/ethnicities (Fig. 1b, c) although there was a strong trend of 44% lower plasma apoE levels in NHW *APOE* ε4 homozygotes compared to ε4 non-carriers (*p* = 0.110, Tukey HSD) (Fig. 1c). However, if solely comparing the plasma apoE levels between NHW *APOE* ε4 non-carriers and homozygous ε4-carriers, the difference in the plasma apoE levels became significant (*p* = 0.045, Student’s *t*-test).

Despite the small sample size, we further noted 2.6 times higher levels of plasma apoE in B/AA *APOE* ε4/ε4 subjects (*n* = 4) compared to NHWs with the same genotype (*n* = 8, Mann–Whitney *U*-test, *p* = 0.017). Due to the low number of study subjects with the *APOE* ε2/ε4 genotype, we did not perform any group comparison between B/AA (*n* = 4) and NHW (*n* = 1).

To account for the potential effects of co-morbidities on plasma apoE levels, we first assessed whether there was any difference in the occurrence of coronary artery disease (CAD), congestive heart failure (CHF), hypertension, hyperlipidemia, atrial fibrillation (Afib), diabetes, stroke/transient ischemic attack (TIA), cancer, chronic renal failure, and chronic obstructive pulmonary disease (COPD) between the diagnostic groups (controls, AD, and MCI patients) and between study subjects that were male/females, BAA/NHW and *APOE* ε4 carriers versus non-carriers. Males exhibited a higher occurrence of CAD (*p* = 0.034), cancer (*p* = 0.025), and diabetes (*p* = 0.05) compared to females. Diabetes (*p* < 0.001) and hypertension (*p* = 0.005) were more common in the BAA subjects whereas Afib was more common in the NHW subjects (*p* = 0.005). As expected, hyperlipidemia was more common in *APOE* ε4-carriers (*p* = 0.009). A diagnosis of hyperlipidemia and statin treatment did not affect plasma apoE levels (Mann–Whitney *U*-test, *p* = 0.922) even when accounting for race/ethnicity (B/AA: Mann–Whitney *U*-test,* p* = 0.928, NHW: Student’s *t*-test, *p* = 0.849), and *APOE* genotype. Similar results were obtained after comparing plasma apoE levels between subjects with and without the listed comorbidities across the cognitive diagnostic groups, sex, and race/ethnicity (data not shown). Scores on the state and national deprivation index, a measurement of how disadvantageous a block area is [38], differed between B/AAs and NHWs (State Deprivation Index: B/AAs median (min–max): 3 (1–10), NHWs median (min–max): 2 (1–8), *p* = 0.002, Mann–Whitney *U*-test, National Deprivation Index: B/AAs median (min–max): 45 (6–100), NHWs median (min–max): 32 (1–78), *p* = 0.001, Mann–Whitney *U*-test); however, plasma apoE levels were not significantly associated with deprivation index scores in any group.

### Plasma apoE isoform composition in *APOE* heterozygous individuals

**Fig. 2:**
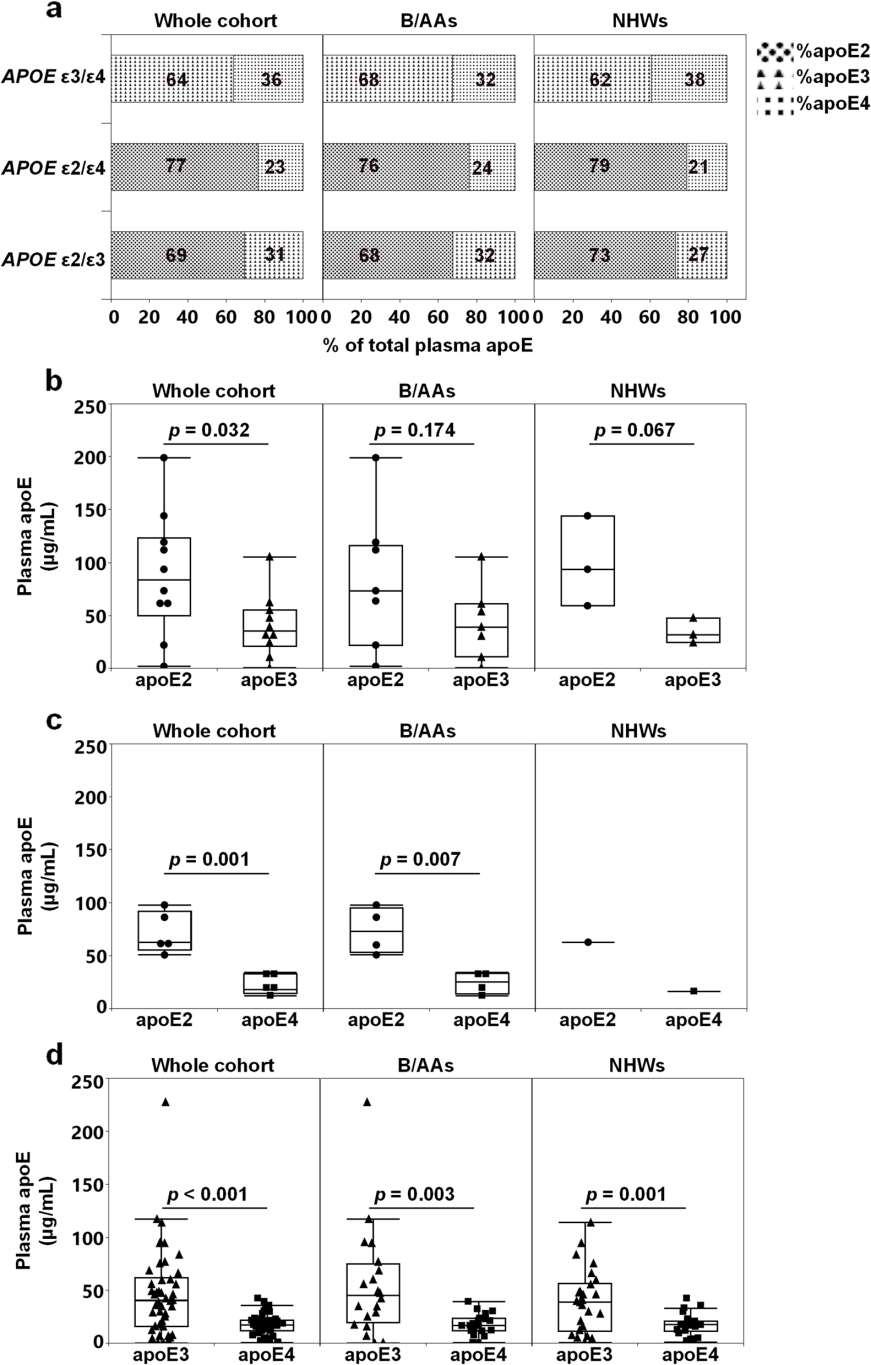
Distribution of *APOE* isoforms in the plasma. ApoE plasma isoform composition as a percentage of total apoE (**a**), plasma concentrations (**b**–**d**) of apoE2 (black dots), apoE3 (black triangles), and apoE4 (black squares) in *APOE* ε2/ε3 (**b**), *APOE* ε2/ε4 (**c**), and *APOE* ε3/ε4 (**d**) subjects. Data is presented % (a) and as medians- with lower and upper quartiles and whiskers indicating the data range (b-d). *p*-values illustrated in **b** and **c** were obtained using the Student’s *t*-test, whereas in **d**, both the Mann–Whitney *U*-test for the comparison in the combined races/ethnicities and Student’s *t*-test for the comparison in Black/African-Americans (B/AAs) and non-Hispanic whites (NHWs) were utilized. Comparison between apoE2 and apoE4 isoforms in *APOE* ε2/ε4 NHW could not be performed due to the low sample number (*n* = 1)

The utilization of LC–MS enabled us to quantify the individual apoE isoforms in order to assess their contribution to the total plasma apoE levels in *APOE* heterozygotes. Previous studies have documented that the plasma apoE isoform composition in heterozygous individuals varies between *APOE* genotypes and that it is rarely equal (50% of each isoform) [10, 14, 15, 32]. Similarly, we here recorded that the apoE2 isoform is the predominant isoform in the plasma from *APOE* ε2 heterozygous subjects and that the apoE3 isoform is the most abundant in *APOE* ε3/ε4 subjects (Fig. 2). The distribution of plasma apoE2 and apoE3 in *APOE* ε2/ε3 subjects was similar across ethnicities with the apoE2 isoform accounting for 67.7 ± 4.4% and 73.1 ± 7.4%, respectively, of the total plasma apoE in B/AAs (*n* = 7) and NHWs (*n* = 3) (Fig. 2a). Similar observations were made in individuals with the *APOE* ε2/ε4 genotype (Fig. 2a). In the whole cohort, levels of the apoE2 isoform were significantly higher compared to the non-apoE2 isoform (either apoE3 or apoE4) (Fig. 2b, c), while in *APOE* ε3/ε4 subjects, levels of the apoE3 isoform were higher compared to the apoE4 isoform (Fig. 2d). The difference in the levels of apoE isoforms remained even after separating the subjects based on their race/ethnic background (Fig. 2b–d). However, the total plasma apoE isoform composition in *APOE* ε3/ε4 subjects did not vary between race/ethnicities (Fig. 2d). Overall, the plasma apoE isoforms contributed to the total plasma apoE levels in the order apoE2 > apoE3 > apoE4 in *APOE* ε2/ε3, *APOE* ε2/ε4, and *APOE* ε3/ε4 subjects. The contribution of individual apoE isoforms to total apoE levels was not influenced by disease status or race/ethnicity.

### Plasma apoE monomer/dimer profiles

Having previously shown that plasma apoE disulfide dimers are of relevance to AD [16], we here aimed to also assess the existence of plasma apoE in monomers and dimers and to investigate whether the apoE monomer/dimer profile is influenced by *APOE* ε4 heterozygosity, race/ethnicity, and/or AD. The presence of cysteine residue(s) in the apoE3 and apoE2 isoforms allows these isoforms to form disulfide dimeric structures which previously have been documented in the plasma, CSF, and the cortex and hippocampus [16, 39–43]. Using SDS-PAGE under non-reducing conditions followed by western blot analysis, we detected two bands of approximately 43 kDa and 95 kDa corresponding to apoE3-apoA-II heterodimers and apoE3-apoE3 homodimers in all plasma samples from subjects with the *APOE* ε3/ε3 and *APOE* ε3/ε4 genotypes (Additional file 1: Fig. S3a-b). The presence of apoE3-apoA-II heterodimers was also previously observed using apoE and apoA-II-specific antibodies [16]. It was earlier shown that apoE2 in *APOE* ε2/ε2 subjects can form apoA-II-apoE2-apoA-II heterotrimers as well as apoE2 multimers [39]. In the plasma from *APOE* ε2/ε3 and *APOE* ε2/ε4 individuals, we recorded a strong immunoreactive band of slightly higher molecular weight than the band corresponding to apoE2 homodimers, most probably due to the formation of apoE2 multimers (Additional file 1: Fig. S3c-d). In individuals with the *APOE* ε2/ε3 genotype, we detected a very faint band at approximately 55 kDa, after longer exposure (2 min), possibly due to the heterotrimeric formation of apoE2 with two apoA-II molecules (Additional file 1: Fig. S3e). In *APOE* ε2/ε4 subjects, this band was not detected (Additional file 1: Fig. S3f). As expected, disulfide dimers were not found in the *APOE* ε4/ε4 subjects (Additional file 1: Fig. S3g). Lastly, in plasma samples from all individuals, we detected a high-molecular-weight band at 130 kDa possibly corresponding to apoE multimers (Additional file 1: Fig. S3).

Quantification of plasma apoE molecular species revealed that in all the studied subjects, plasma apoE exists mainly as monomers and to a lesser extent as dimers (Additional file 1: Table S4A-C). Regardless of disease status or race/ethnicity (subjects with *APOE* ε2/ε3 and *APOE* ε2/ε4 genotype were excluded from the analysis due to the low sample size), individuals with *APOE* ε3/ε4 (*n* = 46) had significantly more monomers (*p* < 0.001, Mann–Whitney *U*-test) and lower total dimers (*p* < 0.001, Mann–Whitney *U*-test) and homodimers (*p* < 0.001, Mann–Whitney *U*-test) compared to *APOE* ε3/ε3 (*n* = 52). We noted 13% more monomers (*p* = 0.001, Student’s *t*-test) followed by a corresponding decrease in total dimers (*p* = 0.001, Student’s *t*-test) due to less apoE3-apoE3 homodimers (*p* < 0.001, Student’s *t*-test), in *APOE* ε3/ε4 B/AAs (*n* = 20), compared to *APOE* ε3/ε3 B/AAs (*n* = 23). Similar differences in the levels of apoE monomers (*p* < 0.001, Student’s *t*-test), total dimers (*p* < 0.001, Student’s *t*-test), and homodimers (*p* < 0.001, Student’s *t*-test) were found in *APOE* ε3/ε3 NHWs (*n* = 29) compared to NHWs with the *APOE* ε3/ε4 genotype (*n* = 26). We did not find any difference in the distribution of plasma apoE monomers and dimers among *APOE* ε3/ε3 and *APOE* ε3/ε4 subjects across diagnostic groups of both races/ethnicities. Overall, the results from this analysis illustrate that plasma apoE mainly exists as monomers in *APOE* ε3/ε3 subjects, with the presence of the ε4 allele further enhancing the amount of apoE monomers. Disease status or race/ethnicity did not have any impact on the distribution of plasma apoE into monomers and dimers.

### Associations between plasma apoE, cognition, CSF markers, and plasma lipids

In order to investigate potential associations between plasma apoE levels and AD-associated pathological processes, we assessed potential links between plasma apoE levels; age; global cognition (MMSE scores); levels of CSF AD biomarkers Αβ_40_, Αβ_42_, t-tau, and p-tau; ratio of tau/Αβ_42_; and other relevant markers including CSF NfL, sTREM2, and plasma lipid levels. Significant associations between plasma apoE and CSF markers or plasma lipids are illustrated in Tables 3 and 4, respectively, whereas non-significant results are shown in Additional file 1: Tables S5 and S6. In the whole cohort, independent of *APOE* genotype, plasma apoE levels were negatively linked to the tau/Aβ_42_ ratio (Table 3) and positively to the levels of plasma HDL and total cholesterol (Table 4). The correlation between plasma apoE with plasma total cholesterol and HDL remained when accounting for the *APOE* genotype (Table 4). Accounting for the clinical diagnosis, the negative association between the CSF tau/Αβ_42_ ratio and plasma apoE levels remained in controls and MCI patients (Table 3). Controls exhibited a positive association between plasma apoE, cholesterol, and LDL levels (Table 4) which remained when accounting for *APOE* genotype, whereas in AD patients, significant associations were found between apoE, HDL, and the LDL/HDL ratio also when *APOE* genotype was accounted for (Table 4).

**Table 3:** Significant correlations between plasma total apoE levels and CSF AD biomarkers

Race/ethnicity	Studied subjects	Sample number (*n*)	CSF markers (pg/mL)	*APOE* genotype unaccounted for		*APOE* genotype accounted for	
				**Correlation (95% CI)**	***p*-value**	**Correlation (95% CI)**	***p*-value**
**Whole cohort**	All	122	t-tau/Αβ_42_	*ρ* = − 0.184 (− 0.355, − 0.001)	0.042	*r* (119) = − 0.122 (− 0.293, − 0.056)	0.183
	Controls	51	Αβ_42_	*r* = 0.307 (0.034, 0.537)	0.029	*r* (48) = 0.287 (0.012, 0.521)	0.044
			t-tau/Αβ_42_	*ρ* = − 0.346 (− 0.567, − 0.078)	0.013	*r* (48) = − 0.292 (− 0.525, − 0.018)	0.040
	MCI	47	t-tau/Αβ_42_	*ρ* = − 0.295 (− 0.536, − 0.009)	0.044	*r* (44) = − 0.225 (− 0.481, 0.066)	0.132
**B/AAs**	Controls	22	Aβ_40_	*r* = 0.481 (0.075, 0.750)	0.023	*r* (19) = 0.478 (0.071, 0.749)	0.028
		23	Αβ_42_	*r* = 0.460 (0.059, 0.733)	0.027	*r* (20) = 0.459 (0.058, 0.733)	0.032
			t-tau	*ρ* = 0.608 (0.262, 0.815)	0.002	*r* (20) = 0.625 (0.287, 0.824)	0.002
	AD	9	Aβ_40_	*r* = − 0.722 (− 0.937, − 0.111)	0.028	*r* (6) = − 0.759 (− 0.946, − 0.191)	0.029
**NHWs**	Controls	28	t-tau/Αβ_42_	*ρ* = − 0.602 (− 0.796, − 0.296)	< 0.001	*r* (25) = − 0.573 (− 0.779, − 0.255)	0.002
			p-tau	*r* = − 0.435 (− 0.695, − 0.074)	0.021	*r* (25) = − 0.399 (− 0.672, − 0.031)	0.039
			t-tau	*r* = − 0.541 (− 0.761, − 0.210)	0.003	*r* (25) = − 0.521 (− 0.749, − 0.184)	0.005

Correlation analysis was performed using Pearson’s (*r*) correlation test or Spearman’s (*ρ*) rank coefficient. Partial correlations are shown as *r* (degrees of freedom) and were obtained after accounting for *APOE* genotype

*CI* Confidence interval, *B/AAs* Black/African-Americans, *NHWs* Non-Hispanic whites, *Αβ*_*40*_, amyloid-β40 peptide, *Αβ*_*42*_ amyloid-β42 peptide, *t-tau* Total tau, *p-tau* Tau phosphorylated at Thr181, *apoE* Apolipoprotein E

**Table 4:** Significant correlations between plasma total apoE and plasma lipids

Race/ethnicity	Studied subjects	Sample number (*n*)	Plasma lipids (mmol/L)	*APOE* genotype unaccounted for		*APOE* genotype accounted for	
				**Correlation (95% CI)**	***p*-value**	**Correlation (95% CI)**	***p*-value**
**Whole cohort**	All	125	t-Ch.	*r* = 0.228 (0.055, 0.388)	0.010	*r* (122) = 0.280 (0.110, 0.434)	0.002
			HDL	*ρ* = 0.187 (0.012, 0.351)	0.037	*r* (122) = 0.414 (0.258, 0.549)	0.002
	Controls	53	t-Ch.	*r* = 0.368 (0.108, 0.581)	0.007	*r* (50) = 0.414 (0.162, 0.615)	0.002
			LDL	*r* = 0.285 (0.016, 0.516)	0.038	*r* (50) = 0.313 (0.047, 0.538)	0.024
	AD	24	HDL	*r* = 0.506 (0.129, 0.755)	0.012	*r* (21) = 0.555 (0.195, 0.783)	0.006
			LDL/HDL	*r* = − 0.460 (− 0.728, − 0.070)	0.024	*r* (21) = − 0.454 (− 0.725, − 0.062)	0.029
**B/AAs**	All	58	t-Ch.	*ρ* = 0.352 (0.104, 0.559)	0.007	*r* (55) = 0.367 (0.121. 0.571)	0.005
			LDL	*ρ* = 0.327 (0.076, 0.539)	0.012	*r* (55) = 0.341 (0.091, 0.550)	0.009
	Controls	25	t-Ch.	*r* = 0.456 (0.074, 0.721)	0.022	*r* (22) = 0.526 (0.165, 0.763)	0.008
			LDL	*r* = 0.414 (0.023, 0.695)	0.040	*r* (22) = 0.464 (0.084, 0.726)	0.022
	AD	9	t-Ch.	*r* = 0.671 (0.012, 0.924)	0.048	*r* (6) = 0.584 (− 0.131, 0.899)	0.128
**NHWs**	All	67	HDL	*ρ* = 0.269 (0.031, 0.478)	0.027	*r* (64) = 0.263 (0.025, 0.473)	0.033
	AD	15	LDL/HDL	*r* = − 0.562 (− 0.834, − 0.070)	0.029	*r* (12) = − 0.462 (− 0.788, 0.066)	0.097

Correlation was assessed using Pearson’s (*r*) correlation test or Spearman’s (*ρ*) rank coefficient. Partial correlations are shown as *r* (degrees of freedom) and were obtained after accounting for *APOE* genotype

*CI* Confidence intervals, *B/AAs* Black/African-Americans, *NHWs* Non-Hispanic whites, *apoE* Apolipoprotein E, *t-Ch.* Total cholesterol, *LDL* Low-density lipoprotein, *HDL* High-density lipoprotein

Looking at potential effects of race/ethnicity, we found positive associations between plasma apoE, CSF Aβ_40_, Aβ_42_, and t-tau (Table 3) as well as total cholesterol and LDL (Table 4) in B/AA control subjects. No associations were found between plasma apoE levels, CSF markers, and lipids in B/AA MCI patients whereas AD patients exhibited a strong negative correlation between plasma apoE and CSF Aβ_40_ (Table 3) and a positive correlation with total cholesterol levels (Table 4). In the NHW controls, we observed a negative correlation between plasma apoE, CSF tau, and p-tau levels and the tau/Aβ_42_ ratio indicating that higher plasma apoE levels would be related to lower CSF tau and p-tau (Table 3). In NHWs, plasma apoE levels were positively associated with plasma HDL levels, whereas only AD patients exhibited a negative relationship between plasma apoE and the LDL/HDL ratio (Table 4). Lastly, when we account for *APOE* genotype and race/ethnicity, the listed associations remained mainly in B/AA subjects and to a lesser extent in NHWs (Tables 3 and 4).

### Isoform-specific correlations between plasma apoE, cognition, lipids, and CSF markers in *APOE* ε3/ε4 subjects

The rather numerous *APOE* ε3/ε4 study participants (*n* = 46) with nearly equal representation in the two racial/ethnic groups allowed for an analysis of apoE isoform-specific associations with cognition, CSF markers, and lipids. The plasma apoE3 and apoE4 levels were significantly and positively correlated with total apoE levels but only correlated with each other in B/AA (Table 5) and not NHW subjects (Pearson’s (*r*) = 0.362, *p* = 0.069). Importantly, we observed no significant correlations between specific plasma apoE3 and apoE4 isoform levels, cognition, nor CSF AD biomarkers. Instead, higher levels of plasma apoE4 correlated positively to plasma total cholesterol and LDL in B/AA subjects only (Table 5), while plasma apoE3 levels instead were near-significantly associated with HDL levels (Spearman’s (*ρ*) = 0.379, *p* = 0.056, *n* = 26) in NHW*APOE* ε3/ε4 subjects only.

**Table 5:** Significant correlations between plasma total apoE, apoE isoforms levels, and plasma lipids in Black/African-American and non-Hispanic white *APOE* ε3/ε4 subjects

Race/ ethnicity	ApoE isoform	t-Ch (mmol/L)	LDL (mmol/L)	Total plasma apoE (μg/mL)	Plasma apoE3 (μg/mL)
Whole cohort (*n* = 46)	apoE3	ns	ns	*ρ* = 0.965 (0.938, 0.980), *p* < 0.001	–
	apoE4	*r* = 0.473 (0.212, 0.671), *p* < 0.001	ns	*r* = 0.597 (0.371, 0.756), *p* < 0.001	*ρ* = 0.529 (0.283, 0.710), *p* < 0.001
B/AAs (*n* = 20)	apoE3	ns	ns	*ρ* = 0.984 (0.960, 0.993), *p* < 0.001	–
	apoE4	*r* = 0.645 (0.283, 0.846), *p* = 0.002	*r* = 0.552 (0.145, 0.799), *p* = 0.012	*ρ* = 0.750 (0.461, 0.895), *p* < 0.001	*ρ* = 0.677 (0.335, 0.861), *p* = 0.001
NHWs (*n* = 26)	apoE3	ns	ns	*r* = 0.958 (0.907, 0.981), *p* < 0.001	–
	apoE4	ns	ns	*r* = 0.605 (0.284, 0.804), *p* < 0.001	ns

Correlation analysis was performed using Pearson’s (*r*) correlation test or Spearman’s (*ρ*) rank coefficient test. Numbers in brackets correspond to 95% confidence intervals

*B/AAs* Black/African-Americans, *NHWs* Non-Hispanic whites, *apoE* Apolipoprotein E, *t-Ch.* Total cholesterol, *LDL* Low-density lipoprotein, *ns* Non-significant

### Associations between plasma apoE monomer/dimer profiles, CSF markers, and lipids

To investigate whether the formation of apoE dimers is related to age, cognition, and AD CSF markers, we assessed potential associations with the occurrence of plasma apoE monomers and dimers*.* Subjects with *APOE* ε2/ε3 and ε2/ε4 genotypes were excluded from the correlation analysis due to low subject numbers*.* As expected, in all the studied subjects with the *APOE* ε3/ε3 and *APOE* ε3/ε4 genotype, we observed an inverse relationship between plasma apoE monomers and total dimers, with the latter also being positively associated with the individual apoE homo- and heterodimers (Additional file 1: Table S7). In addition, apoE3 homodimers and heterodimers were positively associated in *APOE* ε3/ε4 (Pearson’s (*r*) = 0.296, *p* = 0.046, *n* = 46) and B/AAs *APOE* ε3/ε3 (Spearman’s (*ρ*) = 0.413 *p* = 0.050, *n* = 23).

In subjects with the *APOE* ε3/ε3 genotype, we did not document any associations between plasma apoE monomers or dimers, age, cognition, CSF AD biomarkers, CSF NfL, or sTREM2 levels (data not shown), whereas in ε3/ε4 subjects, plasma apoE3 homodimers were positively linked to age (Additional file 1: Table S8) and CSF NfL levels (Pearson’s (*r*) = 0.289, *p* = 0.054, *n* = 46), with the latter not reaching statistical significance.

Separating the subjects into racial/ethnic groups, age was associated with the occurrence of plasma apoE3-apoA-II heterodimer, in NHW *APOE* ε3/ε3 subjects (Additional file 1: Table S8). Age was positively associated with plasma apoE homodimers in NHW *APOE* ε3/ε4 subjects (Additional file 1: Table S8). In the same subjects, we noted a positive association between plasma apoE3 homodimers and CSF levels of Aβ_40_ (Pearson’s (*r*) = 0.498, *p* = 0.011, *n* = 25) and NfL levels (Pearson’s (*r*) = 0.467, *p* = 0.016, *n* = 26).

Specifically looking at individuals with the *APOE* ε3/ε3 and ε3/ε4 genotypes, we found that having more plasma apoE3 monomers was linked to higher plasma triglycerides but lower HDL levels, irrespective of race/ethnicity (Additional file 1: Table S8). Higher amounts of plasma apoE total dimers consequently were linked to plasma triglycerides and HDL levels in the opposite direction. This inverse relationship was exclusively driven by apoE heterodimers (Additional file 1: Table S8). Plasma total cholesterol levels were negatively associated only with the amount of apoE3 homodimers (Additional file 1: Table S8), and the association was not modified by race/ethnicity.

### Plasma lipids and associations with cognition and CSF markers levels

In the whole cohort, higher plasma total cholesterol, HDL, and LDL levels were significantly associated with higher CSF t-tau and p-tau levels, as well as higher sTREM2 regardless of *APOE* genotype, race/ethnicity, or clinical diagnosis (Additional file 1: Table S9).

Combining race/ethnicity with clinical diagnosis revealed similar positive associations in both racial/ethnic groups in regard to plasma total cholesterol, LDL, and HDL with cognition and CSF biomarkers Aβ_40_, Aβ_42_, t-tau, and p-tau (Additional file 1: Table S9). However, specifically in B/AA control subjects, we observed a positive correlation between all the studied plasma lipids and age (Additional file 1: Table S9). In the same group, we also noted that levels of triglycerides were positively linked to CSF NfL levels and negatively to MMSE scores.

## Discussion

The risk of developing AD is approximately two-fold higher in B/AA subjects compared to NHWs [20]. Nevertheless, the AD risk-promoting effect of the *APOE* ε4 genotype, the strongest genetic risk factor for sporadic AD, appears lower in B/AA compared to white individuals [44]. With strong support for a direct association between low plasma apoE levels and an increased risk of AD and other types of dementia [12], we aimed to assess whether the lower ε4-induced risk of AD in B/AAs may be attributed to a different plasma apoE profile than that previously reported in whites [10, 14, 16].

Contrary to studies that have illustrated a higher frequency of ε4 allele in B/AAs [19, 23], no such observation was made in the current cohort, possibly due to the low sample size. In line with previous reports, we found no difference in plasma apoE levels between the diagnostic groups in either racial/ethnic group [10, 13], confirming that plasma apoE levels per se are not suitable as an AD diagnostic biomarker. In line with our hypothesis of B/AA *APOE* ε4-carriers exhibiting a different plasma apoE profile compared to NHWs, potentially underlying their lower *APOE*ε4-promoted risk of AD, we indeed found significantly higher levels of plasma apoE in B/AA *APOE* ε4/ε4-carriers compared to NHWs with the same genotype, despite the very small sample size. There was also no significant difference in plasma apoE levels between *APOE* ε4 non-carriers, heterozygous, and homozygous B/AA subjects whereas there was a strong trend towards lower plasma apoE levels in the NHW *APOE* ε4 homozygous subjects compared to non-carriers, in line with earlier reports [10, 14, 32, 45]. That the latter difference did not reach statistical significance we argue is due to the small sample size.

In both racial/ethnic groups, the apoE4 isoform was less prominent and contributed the least to the total apoE levels in *APOE* ε4 heterozygotes, which is in line with previous studies [10, 13, 15, 32]. Previous studies have reported a higher turnover rate of the apoE4 isoform in plasma, compared to that of apoE2 and apoE3 [46]; however, potential differences in hepatic *APOE* allele expression in heterozygous individuals have yet to be investigated. In the human brain, it was shown that the *APOE ε*4 allele was significantly higher expressed than the non-*APOE ε*4 allele in heterozygous subjects; however, another study suggested significantly higher expression levels of *APOE ε*4 due to polymorphisms in the *APOE* gene promotor [47, 48]. A recent study by Reddy and colleagues reported plasma cell-free mRNA levels of 50 AD-relevant genes, including *APOE*, in B/AA AD patients and cognitively unimpaired controls; however, it remains to be determined how plasma transcripts of *APOE* relate to protein levels of the same. Previous results from Griswold and colleagues proposed the notion of race/ethnicity-driven differences in *APOE* expression showing that the central nervous system expression of *APOE*, specifically in the frontal cortex, differed between *APOE* ε4/ε4 AD patients with African local genomic ancestry versus European local ancestry [28], with significantly higher levels of apoE in the latter group. Based on our findings, we speculate that ancestry-related effects may also influence plasma apoE levels, specifically in *APOE* ε4/ε4 subjects despite the low sample numbers, although our observed effect is in plasma and in the opposite direction to that reported by Griswold et al. A measurement of genomic ancestry on the subjects included in the current cohort was not performed but such an analysis may indeed clarify a potential relationship with plasma apoE levels. Furthermore, we did not find any significant difference in plasma apoE levels between B/AA males and females contrasting previous findings of higher plasma apoE levels in female versus male Caucasian subjects [15]. In NHW females, we did however observe a trend of higher plasma apoE levels compared to males (*p* = 0.062).

In addition to their relevance to dementia, plasma apoE levels have also been implicated in ischemic heart disease [49]. Studies of white individuals have revealed a positive link between elevated levels of plasma apoE and cardiovascular-associated mortality [50] as well as the risk of ischemic heart disease in males [51]. Heart diseases are more frequent in B/AAs compared to NHWs [52], which could be attributed to environmental (i.e., lifestyle, socioeconomic factors) as well as genetic reasons [53, 54] and share similar risk factors with sporadic AD [55], including altered plasma lipids. In our study, B/AAs exhibited higher occurrence of diabetes and hypertension as well as higher state and national deprivation index scores compared to NHWs suggesting that B/AAs were living in a less affluent block area. To our knowledge, no studies have assessed any potential associations between plasma apoE levels and cardiovascular-associated mortality in B/AAs. We did not observe any association between plasma apoE levels and statin treatment, any of the known clinical comorbidities (CAD, CHF, hypertension, hyperlipidemia, cancer, diabetes, Afib, chronic renal failure, COPD, stroke/TIA), or scores on the area deprivation index, which is a better reflection of social economic status than household income as older people may have more assets than incomes.

In our study, B/AA MCI patients exhibited lower plasma triglyceride levels compared to the NHW patients. Higher plasma HDL and lower triglycerides in B/AAs versus white subjects were previously reported and implicated a differential risk of cardiovascular disease in the two groups [56]. That plasma lipid levels are of relevance to neurodegenerative disease processes and AD has been demonstrated in several studies [57]. For instance, higher plasma triglyceride levels at mid-life were linked to AD brain pathology two decades later [58]. A recent lipidomics study of AD patients and healthy subjects further highlighted an altered plasma lipidome alongside AD pathology [59]. In the current study, we observed higher levels of total cholesterol in AD patients compared to MCI patients and controls. Increased total and LDL cholesterol levels were also observed in the studied *APOE* ε4 carriers. High triglyceride levels or low HDL levels were previously associated with cognitive impairment [60–62] and a recent meta-analysis indeed demonstrated a positive link between AD risk and elevated levels of total cholesterol and LDL particles [63]. In our study, we noted that total plasma apoE levels were significantly and positively correlated to plasma LDL levels in the B/AA study subjects whereas plasma apoE levels were significantly associated with HDL in the NHWs. Specifically, plasma apoE4 isoform levels in *APOE* ε3/ε4 subjects were significantly associated with plasma LDL in B/AAs only. Whereas plasma LDL levels are causally associated with vascular disease and atherosclerosis, it appears the protective function of HDL against the same and can be lost [64]. If the lipid composition may vary in an ancestry-dependent manner needs to be established. Importantly, we repeatedly noticed positive correlations between different plasma lipids and CSF tau levels, regardless of clinical diagnosis and *APOE* genotype, indicating an unfavorable association between plasma lipids and tau pathology. Furthermore, gender is well known to influence plasma lipid levels [27], and we also documented, within each racial/ethnic groups, higher levels of cholesterol in females compared to males. Only in B/AAs we further found higher levels of triglycerides in males compared to females. In the B/AA controls, MMSE scores were negatively correlated with triglycerides and positively associated with HDL in support of a link between cognition and the plasma lipid profile.

Aside from a differential risk of AD, differences in the levels of CSF AD biomarkers have been observed between races/ethnicities. Black/African-Americans, including the subjects investigated here, have in several studies exhibited lower CSF levels of t-tau and p-tau compared to white patients, but also lower levels of CSF sTREM2 [33, 65, 66]. We previously reported that low plasma apoE levels were unfavorably linked to cognition and to CSF AD biomarker levels in a sample of ethnic Norwegian subjects from a cohort of longitudinally followed MCI and AD patients [16]. In a recent follow-up study of the same cohort, we in addition discovered a significant correlation between low plasma apoE levels, Aβ brain pathology, and transition from amnestic MCI to AD dementia [32]. In the current study, we found correlations of opposite directions between plasma apoE and CSF t-tau levels in B/AA versus NHW controls, with the latter exhibiting a negative correlation in line with the notion that lower plasma apoE levels may not be beneficial in these subjects [12, 16]. A significant race/ethnicity-by-*APOE*ε4 interaction was previously reported for CSF tau levels [65]; however, whether plasma apoE contributes directly or indirectly to AD risk in ε4 negative B/AA by influencing the levels of CSF tau levels, indicative of brain tau pathology, needs further research. We did not attempt to classify the study subjects based on amyloid/tau/neurodegeneration (A/T/N) since a universal cutoff for mainly t-tau and p-tau may be misleading with B/AA patients reported to exhibit lower CSF t- and p-tau [33, 65, 67, 68]. Furthermore, amyloid-β plaque pathology was proposed to be negatively linked to African ancestry [69]; hence, the development of individualized biomarker cutoff in which race/ethnicity is taken into account, is needed.

The functional relevance of plasma apoE levels in relation to both AD risk and levels of CSF biomarkers is complicated as apoE in subjects with the ε2 and/or ε3 occurs in different monomer, dimer, and multimer formations. In vitro studies have shown that apoE-apoA-II heterodimers have a higher affinity for the Aβ_42_ peptide compared to monomers [70] preventing it from neuronal endocytosis [71]. Furthermore, it was previously shown that in *APOE* ε3/ε3 individuals, apoE dimers account for 55% of total plasma apoE [39]. In our previous study of *APOE* ε3/ε3 subjects included in a longitudinal followed Norwegian cohort, we replicated this finding and further observed that the plasma apoE distribution in monomers and dimers was altered in AD patients [16]. Here, we instead found that approximately 45% of the plasma apoE in *APOE* ε3/ε3 B/AAs and NHWs occurred as dimers and further noted higher amounts of monomers over dimers in subjects with *APOE* ε2/ε3, *APOE* ε2/ε4, and *APOE* ε3/ε4. We also observed that the ratio of apoE monomers/dimers was unrelated to clinical diagnosis and CSF AD biomarkers. Interestingly, previously, it was shown that CSF and brain apoE dimers did not differ between AD patients and cognitively healthy controls [41, 43, 72]. In our earlier work, we however observed that plasma apoE3 homodimers were linked to worse cognition and higher tau/Aβ_42_ ratios in *APOE* ε3/ε3 Norwegian individuals [16], suggesting a positive association between plasma apoE3 homodimers and AD pathology. In the current study, we did not observe any significant correlations between CSF AD biomarkers or cognition with either plasma apoE monomers or dimers; hence, more efforts are needed to elucidate whether specifically less apoE3 dimers in *APOE* ε3 homozygous AD patients replicate in other cohorts [16]. Plasma apoE dimer formation is linked to lipoparticle formation [16] and the observed strong correlations between apoE monomers, dimers, and plasma lipids would support that cohort differences in diet may in part explain the difference in apoE dimers observed in the current study compared to previous studies [16, 39]. Plasma apoE dimers have a lower affinity for the LDL receptor [39, 73], making them less capable of delivering lipids; thus, we speculate that a lipid-enriched western diet might require a higher presence of apoE monomers which may be more efficient in assisting lipid metabolism. Furthermore, we recently showed that plasma apoE3 isoform levels were negatively associated with plasma glucose levels in *APOE* ε3/ ε4 subjects with body mass index (BMI) above 25 [74]. The mechanistic underpinnings of the relationship between plasma apoE3 dimers and glucose levels remain to be investigated, specifically since higher dietary fat intake is associated with impaired glucose tolerance [75].

Black/African Americans are significantly underrepresented in AD research and the limitations of our study in addition to the overall low sample size, include the low number of *APOE* ε2 and *APOE* ε4-carries in both racial/ethnic groups, the rather low number of *APOE* ε4 homozygotes and the complete absence of homozygous *APOE* ε2/ε2 individuals. Due to the low sample size, our results are more prone to type I and II statistical errors. Our study is also limited by the inability to assess whether plasma apoE levels were associated with lifestyle-associated factors like BMI, tobacco, and alcohol use.

## Conclusions

In this study, we have reported plasma apoE levels in a racially diverse cohort including B/AA and NHW patients with AD and MCI, and controls. Our results suggest no differences in total plasma apoE or apoE monomer/dimer profiles between the diagnostic groups; however, significantly higher plasma apoE levels were found in B/AA compared to NHW *APOE* ε4 homozygotes and a strong trend towards *APOE* ε4-driven lower plasma apoE levels in NHWs only. Plasma apoE levels were differentially associated with LDL versus HDL in the racially diverse control groups and the direction of the interaction between plasma apoE and t-tau differed between the groups. Altered plasma apoE levels, potentially due to differential *APOE* expression between racial/ethnic groups, in conjunction with other factors including plasma lipids, may underlie the lower *APOE* ε4-induced risk of AD in B/AAs. Future studies need to address whether the difference in AD risk between B/AAs and NHWs may be driven by additional rare genetic variation in *APOE* and its promotor and which may affect plasma apoE levels [12].

### Supplementary Information

**Additional file 1:** **Table S1.** Spiked amount of the heavy labeled peptides in each sample. **Table S2.** Endogenous peptides in the different *APOE* genotypes. **Table S3.** Mass-spectrometry determined plasma apoE phenotypes. **Table S4A.** Distribution of plasma apoE in monomers, dimers and multimers. **Table S4B.** Plasma apoE monomers/dimer/multimer profile in Black/African-Americans. **Table S4C**. Plasma apoE monomers/dimer/multimer profile in Non-Hispanic whites. **Table S5.** Non-significant correlations between plasma total apoE levels with cognition and CSF AD biomarkers. **Table S6.** Non-significant correlations between plasma total apoE levels and CSF AD biomarkers. **Table S7.** Correlations between plasma apoE monomers and dimers in *APOE* ε3/ε3 and *APOE* ε3/ε4 subjects. **Table S8.** Associations between plasma apoE3 monomers and dimers, plasma lipids and age in *APOE* ε3/ε3 and *APOE* ε3/ε4 subjects. **Table S9.** Significant associations between plasma lipids, age, cognition and CSF markers. **Fig. S1.** Quantification of apoE isoforms. (a) Equation used for the quantification of endogenous apoE peptides LGPLVEQGR, LGADMEDVCGR, LGADMEDVR, LAVYQAGAR and CLAVYQAGAR. (b) Correlation between the peptides LAVYQAGAR and LGPLVEQGR in *APOE* ε3/ε3 (open dots), *APOE* ε3/ε4 (open rhombus) and *APOE* ε4/ε4 (black dots) studied subjects. (c) Correlation between apoE3 isoform levels directly quantified by the peptide LGADMEVCGR or calculated by subtracting the levels of apoE4 peptide LGADMEDVR from the peptide LAVYQAGAR in individuals with *APOE* ε3/ε4 genotype. (d) Correlation between apoE4 isoform levels quantified by the peptide LGADMEVR or calculated by subtracting the levels of apoE3 peptide LGADMEDVCGR from the peptide LAVYQAGAR in subjects with *APOE* ε3/ε4 genotype. (e) Correlation between the levels of plasma apoE quantified by the peptide LGPLVEQGR which is common for all apoE isoforms and by adding the apoE isoforms in individuals with *APOE* ε2/ε3 (open triangles), *APOE *ε2/ε4 (x-shape), *APOE* ε3/ε3 (open dots), *APOE* ε3/ε4 (open rhombus) and *APOE* ε4/ε4 (black dots). **Fig. S2.** Calibration curves for the peptides LGPLVEQGR (a), LGADMEDVCGR (b), LAVYQAGAR (c), and CLAVYQAGAR (d). Increasing amount of heavy labeled peptide added in a plasma pool containing all apoE variants plotted against the ratio of heavy to the corresponding endogenous variant. The linearity of the curves was assessed by the weighted sum of squares (1/X^2^) in non-log transformed data and both axes are shown in logarithmic scale. **Fig. S3.** Plasma apoE molecular species. (a-d) Western blot analysis under non-reducing conditions to visualize plasma apoE monomers (36 kDa), heterodimers (43 kDa) and homodimers (95 kDa) in the plasma of Black/African-Americans (B/AAs) and Non-Hispanic whites (NHWs) with *APOE* ε3/ε3 (a), *APOE* ε3/ε4 (b) as well as with *APOE* ε2/ε3 (c) and *APOE* ε2/ε4 (d) genotypes. An upper band at 100 kDa corresponding to apoE2 multimeric molecular species was found in *APOE* ε2 plasma (c-d). (e-f) In assayed plasma from *APOE *ε2/ε3 Black/African-Americans (B/AAs) and Non-Hispanic whites (NHWs) a band at 55 kDa corresponding to apoA-II-apoE2-apoA-II heterotrimeric structure was detected when the membrane was imaged for 2 minutes (e), this band was not detected in *APOE* ε2/ε4 plasma (f). (g) Only apoE monomers were detected in *APOE* ε4/ε4 plasma samples. ApoE molecular species were detected using the mouse pan-apoE antibody WUE-4 (Novus Biologicals). Molecular weight was determined using the protein ladder (PL) PageRuler™ Prestained Protein Ladder, 10 to 180 kDa (Thermo Fisher Scientific). Color differences for B/AAs or NHWs correspond to clinical diagnosis, with black color indicating controls, while blue and red correspond to MCI and AD patients. **Fig. S4.** Effect of gender and race/ethnicity on the levels of plasma lipids. (a-d) Levels of plasma total cholesterol (a), HDL (b), LDL (c) and triglycerides (d) in males and females of combined racial/ethnic groups as well as with different races/ethnicities. Data is presented as median (minimum – maximum). *p*-values acquired using Student’s *t*-test. ns: non-significant, B/AA: Black/African-Americans, NHW: Non-Hispanic whites, ^*^:*p*-value for data after log transformation. :*p*-value obtained after including the *APOE* genotype of the studied subjects as a covariate in the analysis.

## Data Availability

Not applicable.

## Abbreviations

AD: Alzheimer’s disease
Afib: Atrial fibrillation
AGC: Automatic gain control target
ANOVA: Analysis of variances
ApoE: Apolipoprotein E
A/T/N: Amyloid/tau/neurodegeneration
Aβ: Amyloid-β
Aβ_40_: Amyloid-β40
Aβ_42_: Amyloid-β42
B/AA: Black/African American
BMI: Body mass index
CAD: Coronary artery disease
CHF: Congestive heart failure
COPD: Chronic obstructive pulmonary disease
CSF: Cerebrospinal fluid
DLB: Dementia with Lewy bodies
DTT: Dithiothreitol
ECL: Enhanced chemiluminescence
EDTA: Ethylenediaminetetraacetic acid
HDL: High-density lipoprotein
HLB: Hydrophilic-lipophilic balance
IAA: Iodoacetamide
LC: Liquid chromatography
LDL: Low-density lipoprotein
MCI: Mild cognitive impairment
MMSE: Mini-mental state examination
MRI: Magnetic resonance imaging
MS: Mass spectrometry
NfL: Neurofilament light
NHW: Non-Hispanic White
P-tau181: Phosphorylated tau at Threonine (Thr) 181
sTREM2: Soluble TREM2
TFA: Trifluoroacetic acid
Thr: Threonine
TIA: Transient ischemic attack
T-tau: Total tau
WHO: World health organization
