# Correction: Plasma apolipoprotein E levels, isoform composition, and dimer profile in relation to plasma lipids in racially diverse patients with Alzheimer’s disease and mild cognitive impairment

**DOI:** 10.1186/s13195-023-01306-6

**Published:** 2023-09-27

**Authors:** Andreas Giannisis, Asma Al-Grety, Henrik Carlsson, Jennifer C. Howell, William T. Hu, Kim Kultima, Henrietta M. Nielsen

**Affiliations:** 1 Department of Biochemistry and Biophysics, Stockholm University, Svante Arrhenius Väg 16B, 114 18 Stockholm, Sweden; 2 Department of Medical Sciences, Clinical Chemistry, Uppsala University, Uppsala, Sweden; 3 Department of Neurology, Emory University, Atlanta, GA USA; 4 Department of Neurology, Rutgers-Robert Wood Johnson Medical School, and Institute for Health, Health Care Policy, and Aging Research, New Brunswick, NJ USA

**Correction: Alz Res Ther 15, 119 (2023)**

**https://doi.org/10.1186/s13195-023-01262-1**

Following publication of the original article [1], the authors corrected an error in Abstract section. The sentence "Total plasma apoE levels were 13% higher in B/AA compared to NHW APOEε4/ε4 subjects and associated with plasma high-density lipoprotein (HDL) in NHW subjects but with low-density lipoprotein levels (LDL) in the B/AA subjects." under Results section of the Abstract was amended to "Total plasma apoE levels were 2.6-fold higher in B/AA compared to NHW APOEε4/ε4 subjects and associated with plasma high-density lipoprotein (HDL) in NHW subjects but with low-density lipoprotein levels (LDL) in the B/AA subjects.".

The original article [1] has been updated.

## References

[1] Giannisis A, Al-Grety A, Carlsson H (2023). Plasma apolipoprotein E levels, isoform composition, and dimer profile in relation to plasma lipids in racially diverse patients with Alzheimer’s disease and mild cognitive impairment. Alz Res Ther.

