# Supplementary material for: Plasma apolipoprotein E levels, isoform composition, and dimer profile in relation to plasma lipids in racially diverse patients with Alzheimer’s disease and mild cognitive impairment
Source: Alzheimers Res Ther. 2023 Jul 3;15:119. doi: 10.1186/s13195-023-01262-1 (PMC10316569; doi:10.1186/s13195-023-01262-1)
Supplement: Supplementary file 1 — Additional file 1: Table S1. Spiked amount of the heavy labeled peptides in each sample. Table S2. Endogenous peptides in the different APOE genotypes. Table S3. Mass-spectrometry determined plasma apoE phenotypes. Table S4A. Distribution of plasma apoE in monomers, dimers and multimers. Table S4B. Plasma apoE monomers/dimer/multimer profile in Black/African-Americans. Table S4C. Plasma apoE monomers/dimer/multimer profile in Non-Hispanic whites. Table S5. Non-significant correlations between plasma total apoE levels with cognition and CSF AD biomarkers. Table S6. Non-significant correlations between plasma total apoE levels and CSF AD biomarkers. Table S7. Correlations between plasma apoE monomers and dimers in APOE ε3/ε3 and APOE ε3/ε4 subjects. Table S8. Associations between plasma apoE3 monomers and dimers, plasma lipids and age in APOE ε3/ε3 and APOE ε3/ε4 subjects. Table S9. Significant associations between plasma lipids, age, cognition and CSF markers. Fig. S1. Quantification of apoE isoforms. (a) Equation used for the quantification of endogenous apoE peptides LGPLVEQGR, LGADMEDVCGR, LGADMEDVR, LAVYQAGAR and CLAVYQAGAR. (b) Correlation between the peptides LAVYQAGAR and LGPLVEQGR in APOE ε3/ε3 (open dots), APOE ε3/ε4 (open rhombus) and APOE ε4/ε4 (black dots) studied subjects. (c) Correlation between apoE3 isoform levels directly quantified by the peptide LGADMEVCGR or calculated by subtracting the levels of apoE4 peptide LGADMEDVR from the peptide LAVYQAGAR in individuals with APOE ε3/ε4 genotype. (d) Correlation between apoE4 isoform levels quantified by the peptide LGADMEVR or calculated by subtracting the levels of apoE3 peptide LGADMEDVCGR from the peptide LAVYQAGAR in subjects with APOE ε3/ε4 genotype. (e) Correlation between the levels of plasma apoE quantified by the peptide LGPLVEQGR which is common for all apoE isoforms and by adding the apoE isoforms in individuals with APOE ε2/ε3 (open triangles), APOE ε2/ε4 (x-shape), APOE ε3/ε3 (open dots), APO [file 13195_2023_1262_MOESM1_ESM.docx]

**SUPPLEMENTARY INFORMATION**

**Plasma apolipoprotein E levels, isoform composition and dimer profile in relation to plasma lipids in racially diverse patients with Alzheimer’s disease and mild cognitive impairment**

Andreas Giannisis^a^, Asma Al-Grety^b^, Henrik Carlsson^b^ , Jennifer C. Howell^c^, William T. Hu^c,d^, Kim Kultima^b^, Henrietta M. Nielsen^a,*^

^a^*Department of Biochemistry and Biophysics, Stockholm University, Stockholm, Sweden*

^b^*Department of Medical Sciences, Clinical Chemistry, Uppsala University, Uppsala, Sweden*

*^c^Department of Neurology, Emory University, Atlanta, GA, USA.*

*^d^Department of Neurology, Rutgers-Robert Wood Johnson Medical School, and Institute for Health, Health Care Policy, and Aging Research, New Brunswick, NJ, USA.*

*Correspondence: [henrietta.nielsen@dbb.su.se](mailto:henrietta.nielsen@dbb.su.se)

The supplementary information includes nine tables (Supplementary table 1-9), and four figures (Supplementary fig. 1-4).

**Supplementary table 1:** Spiked amount of the heavy labeled peptides in each sample.

| Endogenous sequence | Internal standards | ApoE isoform | Spiked amount (fmoles) |
| --- | --- | --- | --- |
| _181_LGPLVEQGR_189_ | LGPLVEQGR* | apoE2, apoE3 and apoE4 | 175 |
| _159_LAVYQAGAR_167_ | LAVYQAGAR* | apoE3 and apoE4 | 175 |
| _158_CLAVYQAGAR_167_ | CLAVYQAGAR* | apoE2 | 175 |
| _104_LGADMEDVCGR_114_ | LGADMEDVCGR* | apoE2 and apoE3 | 175 |
| _104_LGADMEDVR_112_ | LGADMEDVR* | apoE4 | 105 |

apoE: apolipoprotein E, apoE2: apolipoprotein E2, apoE3: apolipoprotein E3,

apoE4: apolipoprotein E4.

**Supplementary table 2:** Endogenous peptides in the different *APOE* genotypes.

| *APOE* genotype | Endogenous peptide |
| --- | --- |
| *APOE* ε2/ε3 | LAVYQAGAR, CLAVYQAGAR & LGADMEDVCGR |
| *APOE* ε2/ε4 | LAVYQAGAR, CLAVYQAGAR, LGADMEDVCGR & LGADMEDVR |
| *APOE* ε3/ε4 | LAVYQAGAR, LGADMEDVCGR & LGADMEDVR |
| *APOE* ε3/ε3 | LAVYQAGAR & LGADMEDVCGR |
| *APOE* ε4/ε4 | LAVYQAGAR & LGADMEDVR |

*APOE*: Apolipoprotein E gene.

**Supplementary table 3:** Mass-spectrometry determined plasma apoE phenotypes.

| Race/ethnicity & Diagnosis | | *APOE* ε2/ε3 (*n*) | *APOE* ε2/ε4 (n) | *APOE* ε3/ε3 (*n*) | *APOE* ε3/ε4 (*n*) | *APOE* ε4/ε4 (*n*) |
| --- | --- | --- | --- | --- | --- | --- |
| B/AAs  (*n* = 58) | | 7 | 4 | 23 | 20 | 4 |
| Controls  (*n* = 25) | 6 | 2 | 12 | 5 | - |  |
| MCI  (*n* = 24) | – | 2 | 11 | 9 | 2 |  |
| AD  (*n* = 9) | 1 | – | – | 6 | 2 |  |
| NHWs  (*n* = 67) | | 3 | 1 | 29 | 26 | 8 |
| Controls  (*n* = 28) | 1 | – | 16 | 10 | 1 |  |
| MCI  (*n* = 24) | 2 | – | 9 | 10 | 3 |  |
| AD  (*n* = 15) | – | 1 | 4 | 6 | 4 |  |

B/AAs: Black/African-Americans, NHWs: Non-Hispanic whites, MCI: mild cognitive impairment, AD: Alzheimer’s disease, *APOE*: Apolipoprotein E gene.

**Supplementary table 4A:** Distribution of plasma apoE in monomers, dimers and multimers

| *APOE* genotype |  | Clinical diagnosis | | |
| --- | --- | --- | --- | --- |
| *APOE* ε2/ε3 | **Whole cohort** **(*n* = 10)** | **Controls (*n* = 7)** | **MCI (*n* = 2)** | **AD (*n* = 1)** |
| % apoE2 monomers | 65.4 ± 17.5 | 64.5 ± 18.9 | 75.5 ± 14.8 | 51.1 |
| % apoE2 total multimers^#^ | 34.6 ± 17.5 | 35.5 ± 18.9 | 24.5 ± 14.8 | 48.9 |
| % apoE2-apoAII | 14.9 ± 12.5 | 16.4 ± 14.6 | 8.5 ± 5.5 | 17.5 |
| % apoE2-apoE2 | 6.8 ± 3.5 | 6.7 ± 3.3 | 4.7 ± 3.5 | 11.7 |
| % apoE2-multimer | 12.9 ± 3.51 | 12.4 ± 2.0 | 11.3 ± 5.9 | 19.7 |
| *APOE* ε2/ε4 | **Whole cohort** **(*n* = 5)** | **Controls (*n* = 2)** | **MCI (*n* = 2)** | **AD (*n* = 1)** |
| % apoE2 monomers | 95.7 ± 1.7 | 94.4 ± 2.2 | 96.2 ± 0.5 | 97.1 |
| % apoE2 total multimers^#^ | 4.3 ± 1.7 | 5.6 ± 2.2 | 3.8 ± 0.5 | 2.9 |
| % apoE2-apoAII | 0.6 ± 0.2 | 0.6 ± 0.4 | 0.7 ± 0.1 | 0.5 |
| % apoE2-apoE2 | 1.8 ± 0.7 | 2.3 ± 1.1 | 1.6 ± 0.01 | 1.1 |
| % apoE2- multimer | 1.9 ± 0.8 | 2.7 ± 0.7 | 1.5 ± 0.6 | 1.3 |
| *APOE* ε3/ε3 | **Whole cohort** **(*n* = 52)** | **Controls (*n* = 28)** | **MCI (*n* = 20)** | **AD (*n* = 4)** |
| % apoE3 monomers | 54.6 ± 14.5 | 53.0 ± 15.7 | 56.5 ± 13.1 | 56 ± 15.7 |
| % apoE3 total dimers | 45.4 ± 14.5 | 47.0 ± 15.7 | 43.5 ± 13.1 | 44 ± 15.7 |
| % apoE3-apoAII | 19.6 ± 12.4 | 20.5 ± 14.0 | 18.1 ± 9.8 | 20.3 ± 14.8 |
| % apoE3-apoE3 | 25.8 ± 7.2 | 26.5 ± 7.7 | 25.4 ± 6.6 | 23.7 ± 6.9 |
| *APOE* ε3/ε4 | **Whole cohort** **(*n* = 46)** | **Controls (*n* = 15)** | **MCI (*n* = 19)** | **AD (*n* = 12)** |
| % apoE3 monomers | 70.0 ± 12.6 | 72.2 ± 12.7 | 70.0 ± 10.2 | 67.3 ± 16.1 |
| % apoE3 total dimers | 30.0 ± 12.6 | 27.8 ± 12.7 | 30.0 ± 10.2 | 32.7 ± 16.1 |
| % apoE3-apoAII | 16.5 ± 11.1 | 14.9 ± 11.4 | 16.6 ± 10.5 | 18.3 ± 12.2 |
| % apoE3-apoE3 | 13.5 ± 5.1 | 12.9 ± 4.7 | 13.4 ± 4.3 | 14.4 ± 6.8 |

**Supplementary table 4B:** Plasma apoE monomers/dimer/multimer profile in Black/African-Americans.

| *APOE* genotype |  | Clinical diagnosis | | |
| --- | --- | --- | --- | --- |
| *APOE* ε2/ε3 | **Whole cohort (*n* = 7)** | **Controls (*n* = 6)** | **MCI (*n* = 0)** | **AD (*n* = 1)** |
| % apoE2 monomers | 60.8 ± 18.6 | 62.5 ± 19.8 | – | 51.1 |
| % apoE2 total multimers^#^ | 39.2 ± 18.6 | 37.5 ± 19.8 | – | 48.9 |
| % apoE2-apoAII | 17.9 ± 14.0 | 17.9 ± 15.3 | – | 17.5 |
| % apoE2-apoE2 | 7.7 ± 3.9 | 7.0 ± 3.6 | – | 11.7 |
| % apoE2-multimer | 13.6 ± 3.3 | 12.6 ± 2.1 | – | 19.7 |
| *APOE* ε2/ε4 | **Whole cohort** **(*n* = 4)** | **Controls (*n* = 2)** | **MCI (*n* = 2)** | **AD (*n* = 0 )** |
| % apoE2 monomers | 95.3 ± 1.7 | 94.4 ± 2.2 | 96.2 ± 0.5 | – |
| % apoE2 total multimers^#^ | 4.7 ± 1.7 | 5.6 ± 2.2 | 3.8 ± 0.5 | – |
| % apoE2-apoAII | 0.7 ± 0.2 | 0.6 ± 0.4 | 0.7 ± 0.1 | – |
| % apoE2-apoE2 | 1.9 ± 0.7 | 2.3 ± 1.1 | 1.6 ± 0.01 | – |
| % apoE2- multimer | 2.1 ± 0.85 | 2.7 ± 0.7 | 1.5 ± 0.6 | – |
| *APOE* ε3/ε3 | **Whole cohort** **(*n* = 23)** | **Controls (*n* = 12)** | **MCI (*n* = 11)** | **AD (*n* = 0)** |
| % apoE3 monomers | 55.0 ±15.0 | 56.7 ± 15.1 | 53.2 ± 15.3 | – |
| % apoE3 total dimers | 45.0 ± 15.0 | 43.3 ± 15.1 | 46.8 ± 15.3 | – |
| % apoE3-apoAII | 20.3 ± 12.0 | 19.5 ± 13.1 | 21.1 ± 11.3 | – |
| % apoE3-apoE3 | 24.7 ± 6.4 | 23.8 ± 6.3 | 25.7 ± 6.6 | – |
| *APOE* ε3/ε4 | **Whole cohort** **(*n* = 20)** | **Controls (*n* = 5)** | **MCI (*n* = 9)** | **AD (*n* = 6)** |
| % apoE3 monomers | 69.8 ± 12.7 | 75.3 ± 9.9 | 66.3 ± 11.9 | 69.8 ± 16.1 |
| % apoE3 total dimers | 30.2 ± 12.7 | 24.7 ± 9.9 | 33.4 ± 11.9 | 30.2 ± 16.1 |
| % apoE3-apoAII | 17.1 ± 10.3 | 13.8 ± 8.7 | 20.6 ± 11.7 | 14.7 ± 9.1 |
| % apoE3-apoE3 | 13.1 ± 6.0 | 10.9 ± 4.1 | 12.8 ± 4.9 | 15.5 ± 8.7 |

**Supplementary table 4C:** Plasma apoE monomers/dimer/multimer profile in Non-Hispanic whites.

| *APOE* genotype |  | Clinical diagnosis | | |
| --- | --- | --- | --- | --- |
| *APOE* ε2/ε3 | **Whole cohort (*n* = 3)** | **Controls (*n* = 1)** | **MCI (*n* = 2)** | **AD (*n* = 0)** |
| % apoE2 monomers | 75.9 ± 10.5 | 76.7 | 75.5 ± 14.8 | – |
| % apoE2 total multimers^#^ | 24.1 ± 10.5 | 23.3 | 24.5 ± 14.8 | – |
| % apoE2-apoAII | 8.0 ± 4.0 | 7.0 | 8.5 ± 5.5 | – |
| % apoE2-apoE2 | 4.9 ± 2.5 | 5.3 | 4.7 ± 3.5 | – |
| % apoE2 multimer | 11.2 ± 4.1 | 11.0 | 11.3 ± 5.9 | – |
| *APOE* ε2/ε4 | **Whole cohort (*n* = 1)** | **Controls (*n* = 0)** | **MCI (*n* = 0)** | **AD (*n* = 1)** |
| % apoE2 monomers | 97.1 | – | – | 97.1 |
| % apoE2 total multimers^#^ | 2.9 | – | – | 2.9 |
| % apoE2-apoAII | 0.5 | – | – | 0.5 |
| % apoE2-apoE2 | 1.1 | – | – | 1.1 |
| % apoE2- multimer | 1.3 | – | – | 1.3 |
| *APOE* ε3/ε3 | **Whole cohort** **(*n* = 29)** | **Controls (*n* = 16)** | **MCI (*n* = 9)** | **AD (*n* = 4)** |
| % apoE3 monomers | 54.3 ± 14.5 | 50.3 ± 16.0 | 60.5 ± 9.0 | 56 ± 15.7 |
| % apoE3 total dimers | 45.7 ± 14.5 | 49.7 ± 16.0 | 39.5 ± 9.0 | 44 ± 15.7 |
| % apoE3-apoAII | 19.0 ± 12.8 | 21.3 ± 15.0 | 14.3 ± 6.2 | 20.3 ± 14.8 |
| % apoE3-apoE3 | 26.7 ± 7.7 | 28.4 ± 8.3 | 25.2 ± 6.9 | 23.7 ± 6.9 |
| *APOE* ε3/ε4 | **Whole cohort** **(*n* = 26)** | **Controls (*n* = 10)** | **MCI (*n* = 10)** | **AD (*n* = 6)** |
| % apoE3 monomers | 70.2 ± 12.8 | 70.6 ± 14.1 | 73.1 ±7.8 | 64.9 ± 17.2 |
| % apoE3 total dimers | 29.8 ± 12.8 | 29.4 ± 14.1 | 26.9 ± 7.8 | 35.1 ± 17.2 |
| % apoE3-apoAII | 16.0 ± 11.8 | 15.4 ± 12.9 | 13.0 ± 8.3 | 21.9 ± 14.6 |
| % apoE3-apoE3 | 13.8 ± 4.2 | 14.0 ± 4.8 | 13.9 ± 3.8 | 13.2 ± 4.6 |

Values are represented as average ± standard deviation, MCI: patients with mild cognitive impairment, AD: patients with Alzheimer’s disease, *APOE*: Apolipoprotein E gene, #: Heterotrimers of apoE2 with two apoA-II particles excluded from the % of total multimers.

**Supplementary table 5:** Non-significant correlations between plasma total apoE levels with cognition and CSF AD biomarkers.

| Race/ethnicity | Studied subjects | Sample number (n) | Variables | Correlation | *p*-value |
| --- | --- | --- | --- | --- | --- |
| Whole cohort | All | 122 | MMSE | *ρ =* - 0.046 | 0.617 |
|  |  | 122 | Αβ_42_ | *ρ =* 0.113 | 0.214 |
|  |  | 112 | Αβ_40_ | *r =* - 0.078 | 0.413 |
|  |  | 122 | t-tau | *ρ =* - 0.079 | 0.390 |
|  |  | 122 | p-tau | *ρ =* - 0.074 | 0.416 |
|  |  | 122 | NfL | *ρ =* - 0.143 | 0.116 |
|  |  | 119 | sTREM2 | *ρ =* - 0.133 | 0.148 |
|  | Controls | 51 | MMSE | *ρ =* - 0.047 | 0.157 |
|  |  | 48 | Αβ_40_ | *ρ =* - 0.037 | 0.802 |
|  |  | 51 | t-tau | *ρ =* - 0.094 | 0.513 |
|  |  | 51 | p-tau | *ρ =* - 0.204 | 0.151 |
|  |  | 51 | NfL | *ρ =* - 0.118 | 0.408 |
|  |  | 49 | sTREM2 | *r =* - 0.216 | 0.137 |
|  | MCI | 47 | MMSE | *ρ =* - 0.055 | 0.7135 |
|  |  | 47 | Αβ_42_ | *ρ =* 0.2405 | 0.1034 |
|  |  | 40 | Αβ_40_ | *r* = 0.040 | 0.807 |
|  |  | 47 | t-tau | *ρ =* - 0.128 | 0.393 |
|  |  | 47 | p-tau | *r* = - 0.140 | 0.348 |
|  |  | 47 | NfL | *ρ =* - 0.050 | 0.740 |
|  |  | 46 | sTREM2 | *r =* - 0156 | 0.301 |
|  | AD | 24 | MMSE | *ρ =* 0.054 | 0.804 |
|  |  | 24 | Αβ_42_ | *ρ =* - 0.221 | 0.300 |
|  |  | 24 | Αβ_40_ | *r* = - 0.296 | 0.156 |
|  |  | 24 | t-tau | *r* = 0.182 | 0.395 |
|  |  | 24 | t-tau / Αβ_42_ | *r* = 0.201 | 0.347 |
|  |  | 24 | p-tau | *r* = 0.147 | 0.493 |
|  |  | 24 | NfL | *ρ =* - 0.357 | 0.086 |
|  |  | 24 | sTREM2 | *r =* 0.212 | 0.320 |
| B/AAs | All | 55 | MMSE | *ρ =* - 0.017 | 0.904 |
|  |  | 55 | Αβ42 | *ρ =* 0.139 | 0.311 |
|  |  | 53 | Αβ40 | *ρ =* 0.173 | 0.217 |
|  |  | 55 | t-tau | *ρ =* 0.197 | 0.150 |
|  |  | 55 | t-tau / Αβ_42_ | *ρ =* - 0.044 | 0.749 |
|  |  | 55 | p-tau | *ρ =* 0.126 | 0.361 |
|  |  | 55 | NfL | *ρ =* - 0.040 | 0.773 |
|  |  | 54 | sTREM2 | *ρ =* 0.057 | 0.681 |
|  | Controls | 23 | MMSE | *ρ =* - 0.049 | 0.824 |
|  |  | 23 | t-tau / Αβ_42_ | *ρ =* - 0.048 | 0.829 |
|  |  | 23 | p-tau | *r* = 0.364 | 0.088 |
|  |  | 23 | NfL | *ρ =* 0.065 | 0.768 |
|  |  | 22 | sTREM2 | *r* = 0.139 | 0.536 |
|  | MCI | 23 | MMSE | *r* = - 0.196 | 0.370 |
|  |  | 23 | Αβ_42_ | *r* = 0.146 | 0.505 |
|  |  | 22 | Αβ_40_ | *r* = 0.199 | 0.374 |
|  |  | 23 | t-tau | *r* = - 0.096 | 0.663 |
|  |  | 23 | t-tau / Αβ_42_ | *r* = - 0.147 | 0.504 |
|  |  | 23 | p-tau | *r* = - 0.068 | 0.759 |
|  |  | 23 | NfL | *r* = -0.029 | 0.900 |
|  |  | 23 | sTREM2 | *r* = - 0.018 | 0.935 |
|  | AD | 9 | MMSE | *ρ =* 0.264 | 0.493 |
|  |  | 9 | Αβ_42_ | *r* = -0.241 | 0.532 |
|  |  | 9 | t-tau | *r* = 0.480 | 0.191 |
|  |  | 9 | t-tau / Αβ_42_ | *r* = 0.551 | 0.124 |
|  |  | 9 | p-tau | *r* = 0.525 | 0.147 |
|  |  | 9 | NfL | *ρ =* -0.650 | 0.058 |
|  |  | 9 | sTREM2 | *r* = 0.146 | 0.709 |
| NHWs | All | 67 | MMSE | *ρ =* - 0.042 | 0.739 |
|  |  | 67 | Αβ_42_ | *ρ =* 0.098 | 0.430 |
|  |  | 59 | Αβ_40_ | *r =* - 0.143 | 0.281 |
|  |  | 67 | t-tau | *ρ =* - 0.147 | 0.236 |
|  |  | 67 | t-tau / Αβ_42_ | *ρ =* - 0.224 | 0.068 |
|  |  | 67 | p-tau | *r =* - 0.106 | 0.395 |
|  |  | 67 | NfL | *ρ =* - 0.174 | 0.159 |
|  |  | 65 | sTREM2 | *ρ =* - 0.128 | 0.311 |
|  | Controls | 28 | MMSE | *ρ =* 0.243 | 0.214 |
|  |  | 28 | Αβ42 | *r* = 0.134 | 0.497 |
|  |  | 26 | Αβ40 | *r* = – 0.313 | 0.120 |
|  |  | 28 | NfL | *r* = – 0.284 | 0.143 |
|  |  | 27 | sTREM2 | *r* = – 0.309 | 0.117 |
|  | MCI | 24 | MMSE | *r* = – 0.048 | 0.825 |
|  |  | 24 | Αβ_42_ | *r* = 0.314 | 0.135 |
|  |  | 18 | Αβ_40_ | *r* = 0.173 | 0.492 |
|  |  | 24 | t-tau | *ρ =* 0.072 | 0.738 |
|  |  | 24 | t-tau / Αβ_42_ | *ρ =* – 0.252 | 0.235 |
|  |  | 24 | p-tau | *r* = 0.007 | 0.973 |
|  |  | 24 | NfL | *ρ =* 0.111 | 0.607 |
|  |  | 23 | sTREM2 | *r* = – 0.062 | 0.779 |
|  | AD | 15 | MMSE | *r =* – 0.181 | 0.520 |
|  |  | 15 | Αβ_42_ | *ρ =* – 0.093 | 0.742 |
|  |  | 15 | Αβ_40_ | *r =* – 0.008 | 0.978 |
|  |  | 15 | t-tau | *r =* 0.274 | 0.324 |
|  |  | 15 | t-tau / Αβ_42_ | *r =* 0.285 | 0.303 |
|  |  | 15 | p-tau | *r =* – 0.084 | 0.767 |
|  |  | 15 | NfL | *r =* – 0.186 | 0.507 |
|  |  | 15 | sTREM2 | *r =* 0.296 | 0.285 |

Correlation analysis of plasma apoE with cognition and CSF AD biomarkers (pg/mL) was performed using Pearson’s (*r*) correlation test or Spearman’s (*ρ*) rank coefficient. B/AAs: Black/African-Americans, NHWs: Non-Hispanic whites, MMSE: Mini-mental-state examination, Αβ_40_: amyloid-β40 peptide, Αβ_42_: amyloid-β42 peptide, t-tau: total tau, p-tau: tau phosphorylated at Thr181, NfL: Neurofilament light chain, sTREM2: soluble Triggering receptor expressed on myeloid cells 2.

**Supplementary table 6:** Non-significant correlations between plasma total apoE levels and plasma lipids.

| Race/ethnicity | Studied subjects | Sample number (n) | Plasma lipids  (mmol/L) | Correlation | *p*-value |
| --- | --- | --- | --- | --- | --- |
| Whole cohort | All | 125 | TGs | *ρ =* – 0.037 | 0.679 |
|  |  |  | LDL | *ρ =* 0.168 | 0.061 |
|  |  |  | LDL / HDL | *ρ =* – 0.061 | 0.501 |
|  | Controls | 53 | TGs | *ρ =* – 0.049 | 0.726 |
|  |  |  | HDL | *ρ =* 0.068 | 0.629 |
|  |  |  | LDL / HDL | *r* = 0.060 | 0.671 |
|  | MCI | 48 | TGs | *ρ =* – 0.073 | 0.623 |
|  |  |  | t-Ch. | *r* = 0.083 | 0.576 |
|  |  |  | HDL | *r* = 0.130 | 0.379 |
|  |  |  | LDL | *r* = 0.015 | 0.922 |
|  |  |  | LDL / HDL | *ρ =* – 0.108 | 0.467 |
|  | AD | 24 | TGs | *r* = – 0.149 | 0.486 |
|  |  |  | t-Ch. | *r* = 0.210 | 0.326 |
|  |  |  | LDL | *r* = – 0.090 | 0.675 |
| B/AAs | All | 58 | TGs | *ρ =* 0.052 | 0.697 |
|  |  |  | HDL | *ρ =* 0.136 | 0.310 |
|  |  |  | LDL / HDL | *ρ =* 0.063 | 0.637 |
|  | Controls | 25 | TGs | *ρ =* 0.101 | 0.631 |
|  |  |  | HDL | *ρ =* 0.007 | 0.975 |
|  |  |  | LDL / HDL | *r* = 0.104 | 0.622 |
|  | MCI | 24 | TGs | *r* = – 0.254 | 0.231 |
|  |  |  | t-Ch. | *r* = 0.009 | 0.966 |
|  |  |  | HDL | *r* = 0.333 | 0.112 |
|  |  |  | LDL | *r* = – 0.115 | 0.592 |
|  |  |  | LDL / HDL | *r* = – 0.285 | 0.177 |
|  | AD | 9 | TGs | *r* = 0.175 | 0.652 |
|  |  |  | HDL | *r* = 0.574 | 0.106 |
|  |  |  | LDL | *r* = 0.594 | 0.092 |
|  |  |  | LDL / HDL | *r* = – 0.253 | 0.511 |
| NHWs | All | 67 | TGs | *ρ =* – 0.118 | 0.340 |
|  |  |  | t-Ch. | *r =* 0.165 | 0.183 |
|  |  |  | LDL | *ρ =* 0.016 | 0.899 |
|  |  |  | LDL / HDL | *r =* – 0.237 | 0.053 |
|  | Controls | 28 | TGs | *r =* – 0.235 | 0.229 |
|  |  |  | t-Ch. | *r =* 0.206 | 0.293 |
|  |  |  | HDL | *r =* 0.163 | 0.408 |
|  |  |  | LDL | *r =* 0.014 | 0.943 |
|  |  |  | LDL / HDL | *r =* – 0.172 | 0.382 |
|  | MCI | 24 | TGs | *r =* 0.240 | 0.259 |
|  |  |  | t-Ch. | *r =* 0.266 | 0.210 |
|  |  |  | HDL | *r =* 0.210 | 0.326 |
|  |  |  | LDL | *r =* 0.142 | 0.509 |
|  |  |  | LDL / HDL | *ρ =* – 0.018 | 0.935 |
|  | AD | 15 | TGs | *r =* – 0.303 | 0.273 |
|  |  |  | t-Ch. | *r =* – 0.158 | 0.574 |
|  |  |  | HDL | *r =* 0.454 | 0.090 |
|  |  |  | LDL | *r =* – 0.462 | 0.083 |

Correlation analysis was performed using Pearson’s (*r*) correlation test or Spearman’s (*ρ*) rank coefficient. B/AAs: Black/African-Americans, NHWs: Non-Hispanic whites, TGs: triglycerides, t-Ch.: total cholesterol, LDL: low density lipoprotein, HDL: high density lipoprotein.

**Supplementary table 7:** Correlations between plasma apoE monomers and dimers in *APOE* ε3/ε3 and *APOE* ε3/ε4 subjects.

| Race/ethnicity | *APOE*  genotype | Plasma apoE molecular species | Dimers | Heterodimers | Homodimers |
| --- | --- | --- | --- | --- | --- |
| Whole cohort | *APOE* ε3/ε3 | Monomers  (*n* = 52) | *ρ* = – 1.000  *p* < 0.001 | *ρ* = – 0.883  *p* < 0.001 | *ρ* = – 0.557  *p* < 0.001 |
|  |  | Dimers  (*n* = 52) | - | *r* = 0.861  *p* < 0.001 | *r* = 0.549  *p* < 0.001 |
|  | *APOE* ε3/ε4 | Monomers  (*n* = 46) | *r* = – 1.000  *p* < 0.001 | *r* = – 0.857  *p* < 0.001 | *r* = – 0.485  *p* < 0.001 |
|  |  | Dimers  (*n* = 46) | - | *ρ* = 0.901  *p* < 0.001 | *r* = 0.486  *p* < 0.001 |
| B/AAs | *APOE* ε3/ε3 | Monomers  (*n* = 23) | *ρ* = – 1.000  *p* < 0.001 | *ρ* = – 0.946  *p* < 0.001 | *ρ* = – 0.607  *p* = 0.002 |
|  |  | Dimers  (*n* = 23) | - | *r* = 0.940  *p* < 0.001 | *ρ* = 0.603  *p* = 0.002 |
|  | *APOE* ε3/ε4 | Monomers  (*n* = 20) | *r* = – 1.000  *p* < 0.001 | *r* = – 0.882  *p* < 0.001 | *r* = – 0.597  *p* = 0.005 |
|  |  | Dimers  (*n* = 20) | - | *r* = 0.882  *p* < 0.001 | *r* = 0.597  *p* = 0.005 |
| NHWs | *APOE* ε3/ε3 | Monomers  (*n* = 29) | *r* = – 1.000  *p* < 0.001 | *ρ* = – 0.850  *p* < 0.001 | *r* = – 0.464  *p* = 0.011 |
|  |  | Dimers  (*n* = 29) | - | *r* = 0.828  *p* < 0.001 | *r* = 0.465  *p* = 0.011 |
|  | *APOE* ε3/ε4 | Monomers  (*n* = 26) | *r* = – 1.000  *p* < 0.001 | *r* = – 0.863  *p* < 0.001 | *r* = – 0.386  *p* = 0.051 |
|  |  | Dimers  (*n* = 26) | - | *r* = 0.918  *p* < 0.001 | *ρ* = 0.387  *p* = 0.051 |

Correlations were determined using Pearson’s (*r*) correlation test or Spearman’s (*ρ*) rank coefficient. B/AAs: Black/African-Americans, NHWs: Non-Hispanic whites, *APOE*: Apolipoprotein E gene.

**Supplementary table 8:** Associations between plasma apoE3 monomers and dimers, plasma lipids and age in *APOE* ε3/ε3 and *APOE* ε3/ε4 subjects.

| Race/ethnicity | *APOE* genotype | Plasma apoE molecular species | | Variables | Sample number (*n*) | Correlation | *p*-vale |
| --- | --- | --- | --- | --- | --- | --- | --- |
| Whole cohort | *APOE* ε3/ε3 | Monomers | | TGs | 52 | *ρ* = 0.764 | < 0.001 |
|  |  |  |  | HDL | 52 | *ρ* = – 0.474 | < 0.001 |
|  |  |  |  | LDL/HDL | 52 | *ρ* = 0.434 | 0.001 |
|  |  | Dimers | | TGs | 52 | *r* = – 0.712 | < 0.001 |
|  |  |  |  | HDL | 52 | *r* = 0.440 | 0.001 |
|  |  |  |  | LDL/HDL | 52 | *r* = – 0.436 | 0.001 |
|  |  | Heterodimers | | TGs | 52 | *r* = – 0.643 | < 0.001 |
|  |  |  |  | HDL | 52 | *r* = 0.582 | < 0.001 |
|  |  |  |  | LDL/HDL | 52 | *r* = – 0.504 | < 0.001 |
|  |  | Homodimers | | TGs | 52 | *r* = – 0.328 | 0.018 |
|  |  |  |  | t-Ch. | 52 | *r* = – 0.314 | 0.023 |
|  | *APOE* ε3/ε4 | Monomers | | TGs | 46 | *r =* 0.642 | < 0.001 |
|  |  |  |  | HDL | 46 | *r =* – 0.523 | < 0.001 |
|  |  |  |  | LDL/HDL | 46 | *r =* 0.438 | 0.002 |
|  |  | Dimers | | TGs | 46 | *ρ = –* 0.599 | < 0.001 |
|  |  |  |  | HDL | 46 | *ρ =* 0.549 | < 0.001 |
|  |  |  |  | LDL/HDL | 46 | *r =* – 0.428 | 0.003 |
|  |  | Heterodimers | | TGs | 46 | *r =* – 0.611 | < 0.001 |
|  |  |  |  | HDL | 46 | *r =* 0.466 | 0.001 |
|  |  |  |  | LDL/HDL | 46 | *r =* – 0.373 | 0.011 |
|  |  | Homodimers | | TGs | 46 | *r =* – 0.354 | 0.016 |
|  |  |  |  | Age | 46 | *r =* 0.371 | 0.011 |
| B/AAs | *APOE* ε3/ε3 | Monomers | | TGs | 23 | *ρ* = 0.660 | 0.002 |
|  |  |  |  | HDL | 23 | *ρ* = – 0.508 | 0.013 |
|  |  |  |  | LDL/HDL | 23 | *ρ* = 0.544 | 0.007 |
|  |  | Dimers | | TGs | 23 | *r* = – 0.626 | 0.001 |
|  |  |  |  | HDL | 23 | *r* = 0.409 | 0.053 |
|  |  |  |  | LDL/HDL | 23 | *r* = – 0.465 | 0.025 |
|  |  | Heterodimers | | TGs | 23 | *r* = – 0.554 | 0.006 |
|  |  |  |  | HDL | 23 | *r* = 0.554 | 0.006 |
|  |  |  |  | LDL/HDL | 23 | *r* = – 0.573 | 0.004 |
|  |  | Homodimers | | TGs | 23 | *ρ* = – 0.450 | 0.031 |
|  | *APOE* ε3/ε4 | Monomers | | TGs | 20 | *r* = 0.660 | 0.002 |
|  |  |  |  | LDL/HDL | 20 | *r* = 0.486 | 0.030 |
|  |  | Dimers | | TGs | 20 | *ρ* = – 0.564 | 0.010 |
|  |  |  |  | LDL/HDL | 20 | *r* = – 0.486 | 0.030 |
|  |  | Heterodimers | | TGs | 20 | *r* = – 0.584 | 0.007 |
|  |  | Homodimers | | TGs | 20 | *r* = – 0.548 | 0.012 |
| NHWs | *APOE* ε3/ε3 | Monomers | | TGs | 29 | *ρ* = 0.824 | <0.001 |
|  |  |  |  | HDL | 29 | *r* = – 0.539 | 0.003 |
|  |  |  |  | LDL/HDL | 29 | *r* = 0.403 | 0.030 |
|  |  | Dimers | | TGs | 29 | *r* = – 0.787 | <0.001 |
|  |  |  |  | HDL | 29 | *r* = 0.537 | 0.003 |
|  |  |  |  | LDL/HDL | 29 | *r* = – 0.403 | 0.030 |
|  |  | Heterodimers | | TGs | 29 | *r* = – 0.689 | <0.001 |
|  |  |  |  | HDL | 29 | *r* = 0.620 | <0.001 |
|  |  |  |  | LDL/HDL | 29 | *ρ* = – 0. 446 | 0.015 |
|  |  |  |  | LDL/HDL | 29 | *ρ* = – 0. 446 | 0.015 |
|  |  | |  | Age | 29 | *r* = – 0.398 | 0.032 |
|  | *APOE* ε3/ε4 | Monomers | | TGs | 26 | *r* = 0.633 | 0.001 |
|  |  |  |  | HDL | 26 | *r* = – 0.613 | <0.001 |
|  |  |  |  | LDL/HDL | 26 | *r* = 0.399 | 0.044 |
|  |  | Dimers | | TGs | 26 | *r* = – 0.609 | <0.001 |
|  |  |  |  | HDL | 26 | *r* = 0.612 | <0.001 |
|  |  |  |  | LDL/HDL | 26 | *r* = – 0.398 | 0.044 |
|  |  | Heterodimers | | TGs | 26 | *r* = – 0.641 | <0.001 |
|  |  |  |  | HDL | 26 | *r* = 0.652 | <0.001 |
|  |  |  |  | LDL/HDL | 26 | *r* = – 0.414 | 0.036 |
|  |  | Homodimers | | Age | 26 | *ρ =* 0.542 | 0.004 |

Correlation analysis of plasma apoE monomers/dimers with age and plasma lipids (mmol/L) was performed using Pearson’s (*r*) correlation test or Spearman’s (*ρ*) rank coefficient. B/AAs: Black/African-Americans, NHWs: Non-Hispanic whites, *APOE*: Apolipoprotein E gene, TGs: triglycerides, t-Ch: total cholesterol, LDL: low density lipoprotein, HDL: high density lipoprotein.

**Supplementary table 9:** Significant associations between plasma lipids, age, cognition and CSF markers.

|  |  |  |  |  | *APOE* genotype unaccounted for | | *APOE* genotype  accounted for | |
| --- | --- | --- | --- | --- | --- | --- | --- | --- |
| Race/ethnicity | **Studied subjects** | **Plasma lipids**  **(mmol/L)** | **Variables** | **Sample number (*n*)** | **Correlation** | ***p*-value** | **Correlation** | ***p*-value** |
| Whole cohort | All | t-Ch. | t-tau | 122 | *r* = 0.178 | 0.050 | *r*(119) = 0.154 | 0.091 |
|  |  |  | p-tau | 122 | *r* = 0.207 | 0.022 | *r*(119) = 0.164 | 0.071 |
|  |  |  | sTREM2 | 119 | *r* = 0.243 | 0.008 | *r*(116) = 0.227 | 0.013 |
|  |  | HDL | t-tau | 122 | *r* = 0.189 | 0.037 | *r*(119) = 0.182 | 0.046 |
|  |  |  | p-tau | 122 | *r* = 0.233 | 0.010 | *r*(119) = 0.226 | 0.013 |
|  |  | LDL | sTREM2 | 119 | *r* = 0.183 | 0.046 | *r*(116) = 0.169 | 0.068 |
|  | Controls | t-Ch. | Αβ_42_ | 51 | *r* = 0.328 | 0.019 | *r*(48) = 0.341 | 0.015 |
|  |  | HDL | MMSE | 51 | *ρ* = 0.302 | 0.031 | *r*(48) = 0.304 | 0.032 |
|  |  |  | t-tau/ Αβ_42_ | 51 | *ρ* = – 0.285 | 0.043 | *r*(48) = – 0.317 | 0.025 |
|  | MCI | t-Ch. | sTREM | 46 | *r* = 0.333 | 0.024 | *r*(43) = 0.340 | 0.022 |
|  |  | TGs | p-tau | 47 | *r* = – 0.290 | 0.048 | *r*(44) = – 0.289 | 0.052 |
|  |  | HDL | t-tau | 47 | *r* = 0.460 | 0.001 | *r*(44) = 0.468 | 0.001 |
|  |  |  | p-tau | 47 | *r* = 0.499 | < 0.001 | *r*(44) = 0.513 | < 0.001 |
|  |  | LDL/HDL | p-tau | 47 | *r* = – 0.344 | 0.018 | *r*(44) = – 0.350 | 0.017 |
|  | AD | TGs | Αβ_42_ | 24 | *r* = – 0.427 | 0.037 | *r*(21) = – 0.443 | 0.034 |
|  |  |  | p-tau | 24 | *r* = 0.512 | 0.011 | *r*(21) = 0.512 | 0.012 |
| B/AAs | All | t-Ch. | sTREM2 | 54 | *r* = 0.396 | 0.003 | *r*(51) = 0.396 | 0.003 |
|  |  |  | t-tau | 55 | *r* = 0.416 | 0.002 | *r*(52) = 0.392 | 0.003 |
|  |  | TG | Age | 58 | *r* = 0.318 | 0.015 | *r*(55) = 0.318 | 0.016 |
|  |  | HDL | p-tau | 55 | *r* = 0.464 | < 0.001 | *r*(52) = 0.422 | 0.001 |
|  |  | LDL | sTREM2 | 54 | *r* = 0.344 | 0.011 | *r*(51) = 0.341 | 0.012 |
|  |  |  | t-tau | 55 | *r* = 0.308 | 0.022 | *r*(55) = 0.292 | 0.032 |
|  | Controls | t-Ch. | Age | 25 | *r* = 0.444 | 0.026 | *r*(22) = 0.435 | 0.034 |
|  |  |  | Αβ_40_ | 22 | *r* = 0.422 | 0.050 | *r*(19) = 0.452 | 0.040 |
|  |  | TGs | Age | 25 | *r* = 0.533 | 0.006 | *r*(22) = 0.547 | 0.006 |
|  |  |  | NFL | 23 | *r* = 0.525 | 0.010 | *r*(20) = 0.609 | 0.003 |
|  |  |  | MMSE | 23 | *ρ* = – 0.429 | 0.041 | *r*(20) = – 0.443 | 0.039 |
|  |  | HDL | MMSE | 23 | *ρ* = 0.429 | 0.041 | *r*(20) = 0.376 | 0.085 |
|  |  | LDL | Age | 25 | *r* = 0.495 | 0.012 | *r*(22) = 0.488 | 0.016 |
|  |  | LDL/HDL | Age | 25 | *r* = 0.585 | 0.002 | *r*(22) = 0.592 | 0.002 |
|  |  |  | MMSE | 23 | *ρ* = – 0.490 | 0.018 | *r*(20) = – 0.530 | 0.011 |
|  | MCI | t-Ch. | sTREM2 | 23 | *r* = 0.460 | 0.027 | *r*(20) = 0.459 | 0.032 |
|  |  | HDL | t-tau | 23 | *r* = 0.439 | 0.036 | *r*(20) = 0.439 | 0.041 |
|  |  |  | p-tau | 23 | *r* = 0.600 | 0.002 | *r*(20) = 0.610 | 0.003 |
|  |  | LDL | sTREM2 | 23 | *r* = 0.420 | 0.046 | *r*(20) = 0.418 | 0.053 |
| NHWs | Controls | t-Ch. | Αβ_42_ | 28 | *r* = 0.389 | 0.041 | *r*(25) = 0.397 | 0.040 |
|  |  |  | t-tau/ Αβ_42_ | 28 | *ρ* = – 0.408 | 0.031 | *r*(25) = – 0.436 | 0.023 |
|  |  | LDL | Αβ_40_ | 28 | *r* = 0.412 | 0.037 | *r*(25) = 0.416 | 0.039 |
|  | MCI | HDL | t-tau | 24 | *r* = 0.511 | 0.011 | *r*(21) = 0.528 | 0.010 |
|  |  |  | p-tau | 24 | *r* = 0.541 | 0.006 | *r*(21) = 0.589 | 0.003 |
|  |  | LDL/HDL | t-tau | 24 | *r* = – 0.566 | 0.004 | *r*(21) = – 0.581 | 0.004 |
|  |  |  | t-tau/ Αβ_42_ | 24 | *ρ* = – 0.435 | 0.034 | *r*(21) = – 0.569 | 0.005 |
|  | AD | TGs | p-tau | 15 | *r* = 0.545 | 0.036 | *r*(12) =0.553 | 0.040 |
|  |  |  | Αβ_42_ | 15 | *ρ* = – 0.652 | 0.008 | *r*(12) = – 0.652 | 0.011 |

Correlation analysis of plasma lipids with age, MMSE scores, CSF biomarkers (pg/mL) was done using Pearson’s (*r*) correlation test or Spearman’s (*ρ*) rank coefficient. Partial correlations are shown as *r* (degrees of freedom) and obtained after accounting for *APOE* genotype. B/AAs: Black/African-Americans, NHWs: Non-Hispanic whites, CSF: cerebrospinal fluid, *APOE*: Apolipoprotein E gene, TGs: triglycerides, t-Ch.: total cholesterol, LDL: low density lipoprotein, HDL: high density lipoprotein, t-tau: total tau, p-tau: tau phosphorylated at threonine 181, sTREM2: soluble TREM2, NfL: neurofilament light chain, MMSE: mini mental state examination score.

**
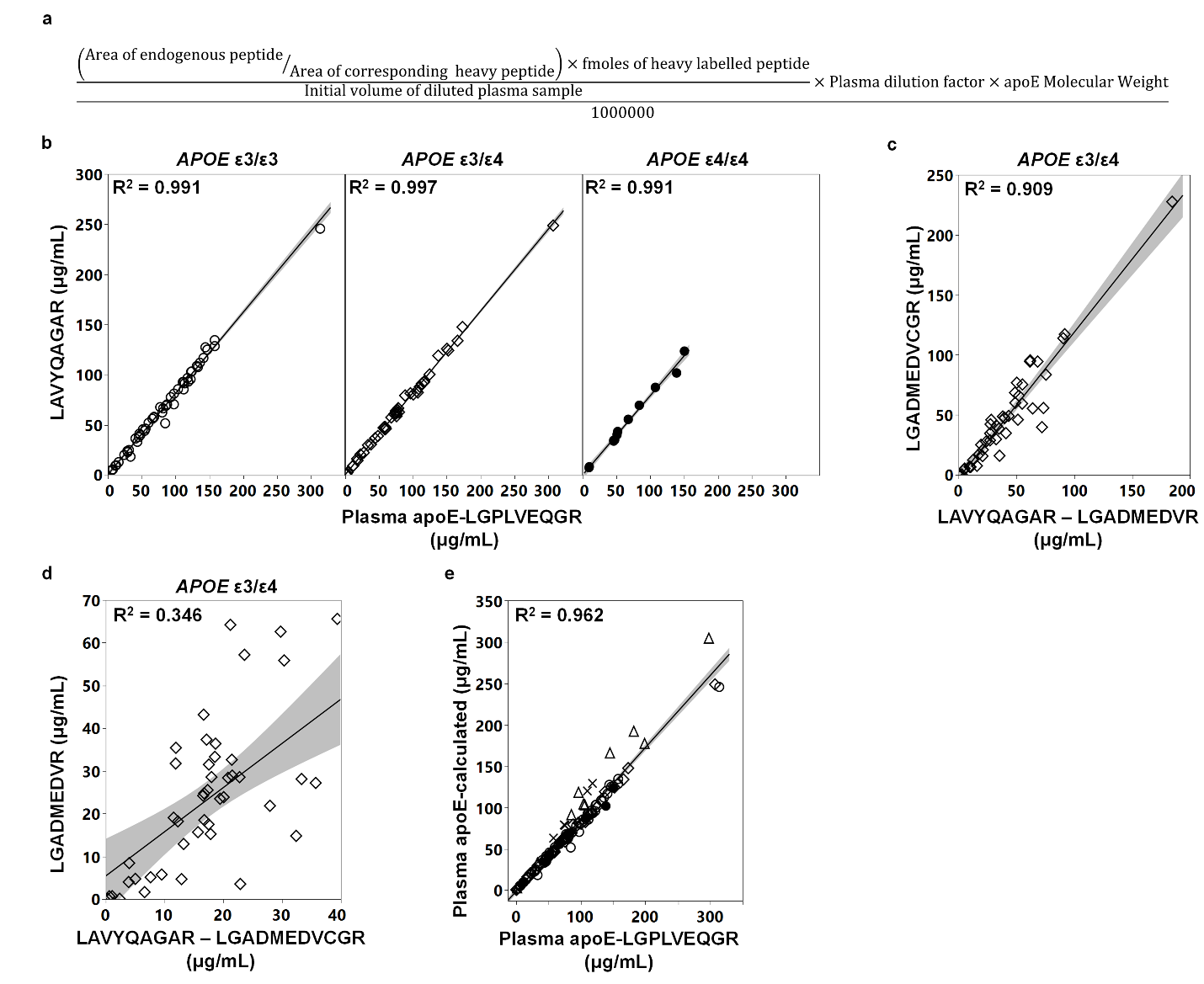
**

**Supplementary figure 1:** Quantification of apoE isoforms.

**(a)** Equation used for the quantification of endogenous apoE peptides LGPLVEQGR, LGADMEDVCGR, LGADMEDVR, LAVYQAGAR and CLAVYQAGAR.

**(b)** Correlation between the peptides LAVYQAGAR and LGPLVEQGR in *APOE* ε3/ε3 (open dots), *APOE* ε3/ε4 (open rhombus) and *APOE* ε4/ε4 (black dots) studied subjects.

**(c)** Correlation between apoE3 isoform levels directly quantified by the peptide LGADMEDVCGR or calculated by subtracting the levels of apoE4 peptide LGADMEDVR from the peptide LAVYQAGAR in individuals with *APOE* ε3/ε4 genotype.

**(d)** Correlation between apoE4 isoform levels quantified by the peptide LGADMEDVR or calculated by subtracting the levels of apoE3 peptide LGADMEDVCGR from the peptide LAVYQAGAR in subjects with *APOE* ε3/ε4 genotype.

**(e)** Correlation between the levels of plasma apoE quantified by the peptide LGPLVEQGR which is common for all apoE isoforms and by adding the apoE isoforms in individuals with *APOE* ε2/ε3 (open triangles), *APOE* ε2/ε4 (x-shape), *APOE* ε3/ε3 (open dots), *APOE* ε3ε4 (open rhombus) and *APOE* ε4/ε4 (black dots).

**
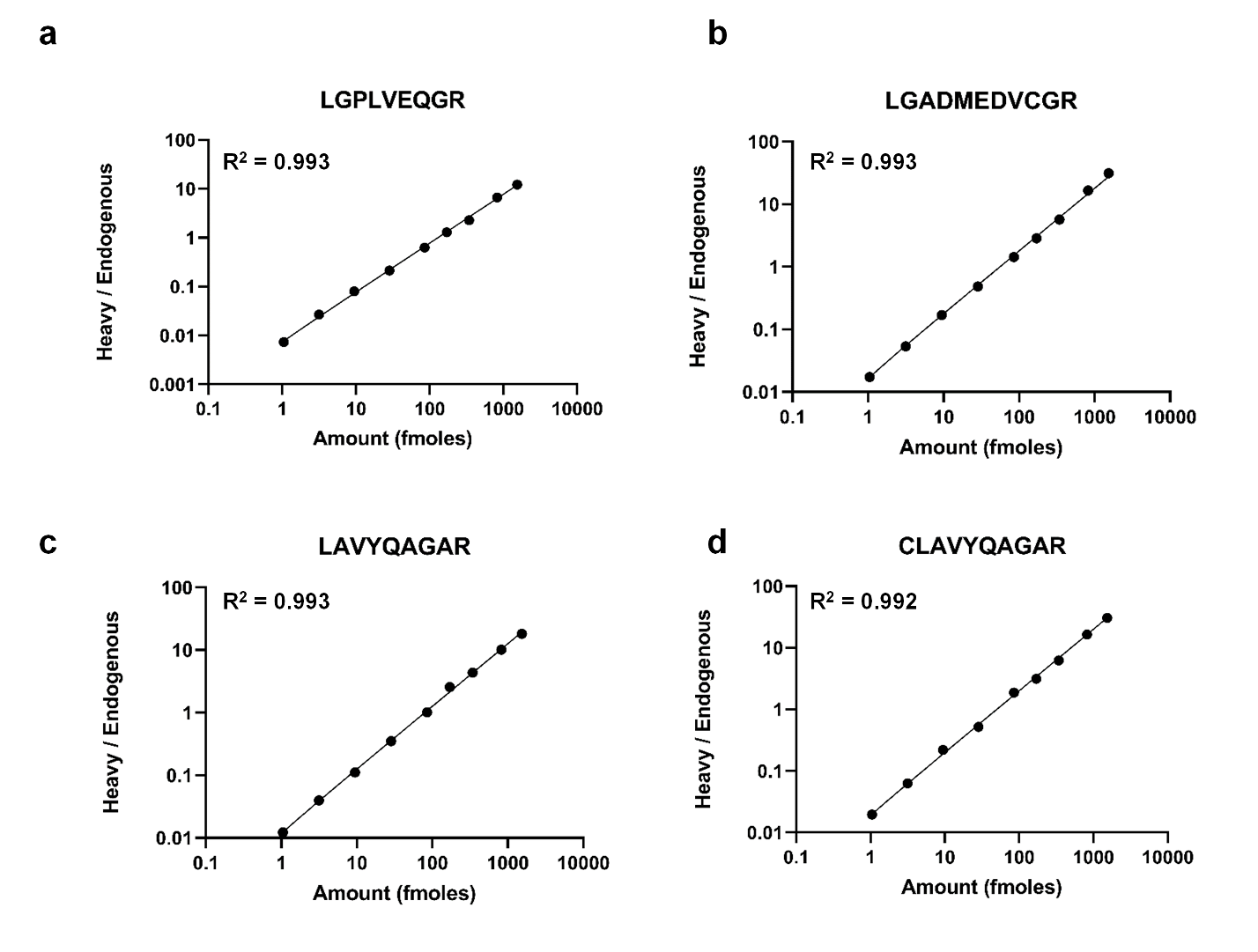
**

**Supplementary figure 2:** Calibration curves for the peptides LGPLVEQGR **(a)**, LGADMEDVCGR **(b)**, LAVYQAGAR **(c)**, and CLAVYQAGAR **(d)**. Increasing amounts of heavy labelled peptide added in a plasma pool containing all apoE variants plotted against the ratio of heavy to the corresponding endogenous variant. The linearity of the curves was assessed by the weighted sum of squares (1/X^2^) in non-log transformed data and both axes are shown in logarithmic scale.


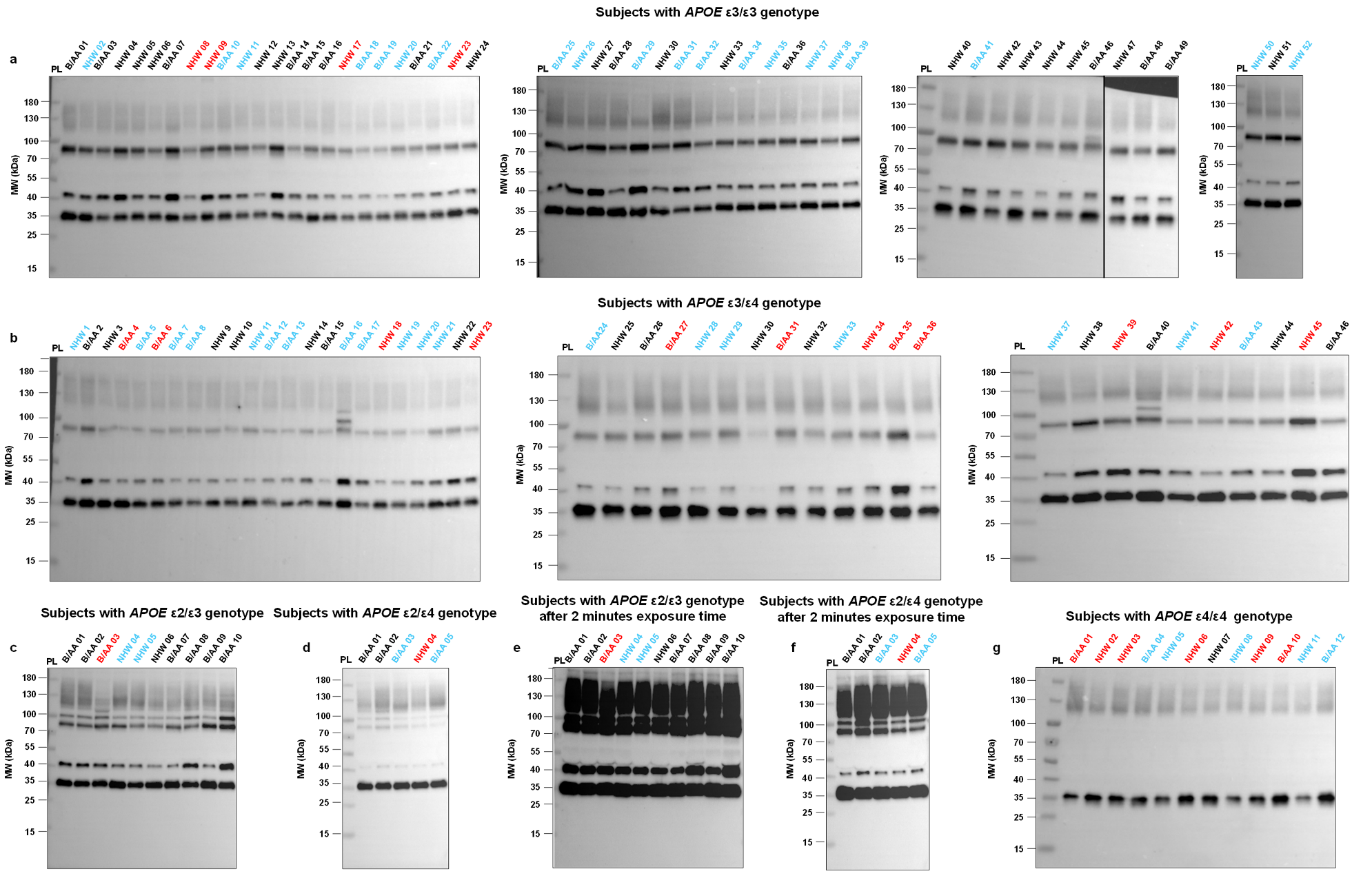


**Supplementary figure 3:** Plasma apoE molecular species.

**(a-d)** Western blot analysis under non-reducing conditions to visualize plasma apoE monomers (36 kDa), heterodimers (43 kDa) and homodimers (95 kDa) in the plasma of Black/African-Americans (B/AAs) and Non-Hispanic whites (NHWs) with *APOE* ε3/ε3 **(a)**, A*POE* ε3/ε4 **(b)** as well as with *APOE* ε2/ε3 **(c)** and *APOE* ε2/ε4 **(d)** genotypes. An upper band at 100 kDa corresponding to apoE2 multimeric molecular species was found in *APOE* ε2 plasma **(c-d)**.

**(e-f)** In assayed plasma from *APOE* ε2/ε3 Black/African-Americans (B/AAs) and Non-Hispanic whites (NHWs) a band at 55 kDa corresponding to apoA-II-apoE2-apoA-II heterotrimeric structure was detected when the membrane was imaged for 2 minutes **(e)**, this band was not detected in *APOE* ε2/ε4 plasma **(f)**.

**(g)** Only apoE monomers were detected in *APOE* ε4/ε4 plasma samples.

ApoE molecular species were detected using the mouse pan-apoE antibody WUE-4 (Novus Biologicals). Molecular weight was determined using the protein ladder (PL) PageRuler™ Prestained Protein Ladder, 10 to 180 kDa (ThermoFisher Scientific). Color differences for B/AAs or NHWs correspond to clinical diagnosis, with black color indicating controls, while blue and red correspond to MCI and AD patients.

**
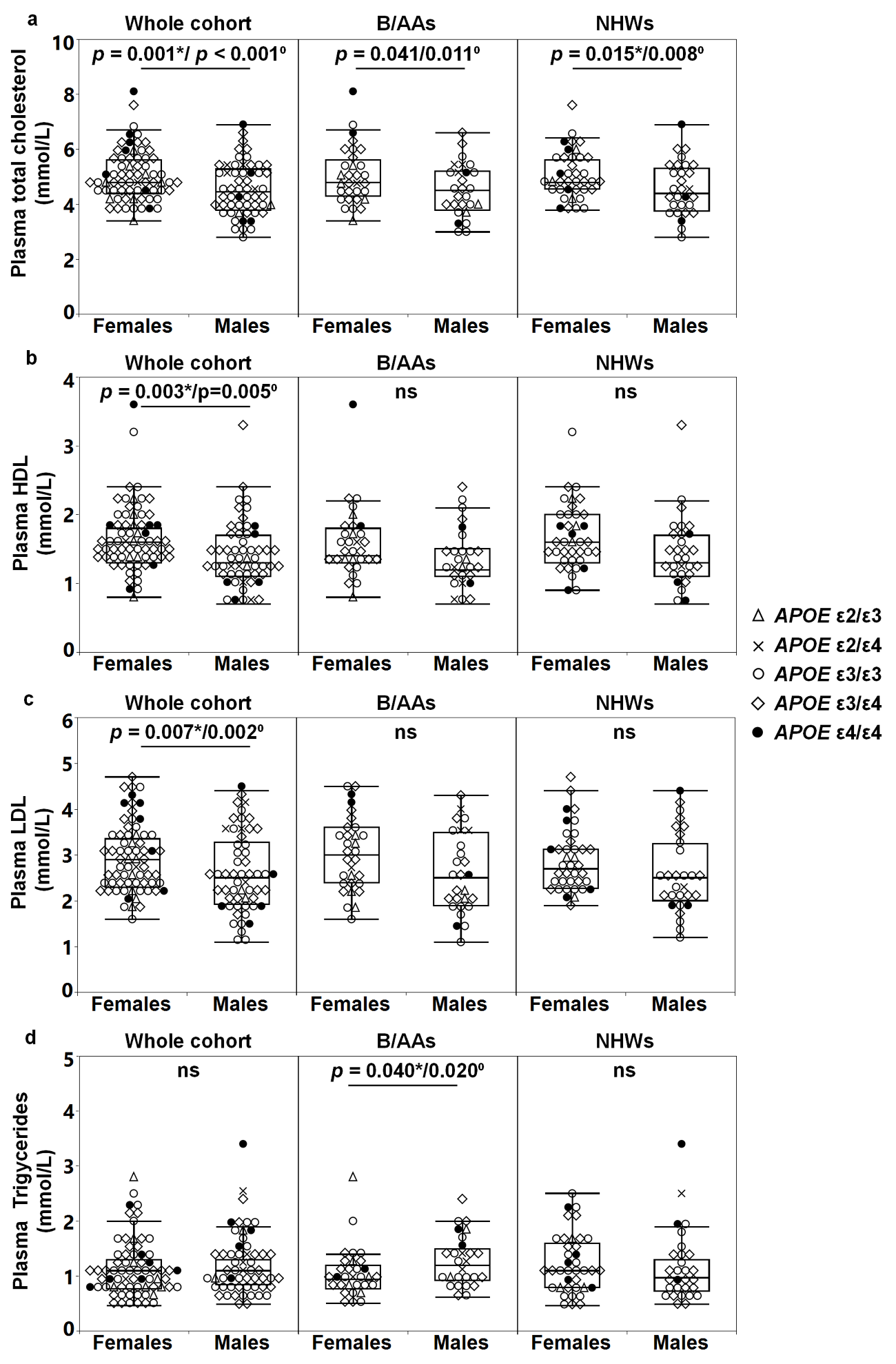
**

**Supplementary figure 4:** Effect of gender and race/ethnicity on the levels of plasma lipids.

**(a-d)** Levels of plasma total cholesterol **(a)**, HDL **(b),** LDL **(c)** and triglycerides **(d)** in males and females of combined racial/ethnic groups as well as with different races/ethnicities. Data is presented as in boxplots showing the median, lower and upper quartiles and with whiskers indicating the data range. *p*-values acquired using Student’s *t*-test. ns: non-significant, B/AAs: Black/African-Americans, NHWs: Non-Hispanic whites, ^*^:*p*-value for data after log transformation. ^⁰^:*p*-value obtained after including the *APOE* genotype of the studied subjects as a covariate in the analysis
